# A study on Electrical Discharge Machining of Titanium Grade2 with experimental and theoretical analysis

**DOI:** 10.1038/s41598-021-88534-8

**Published:** 2021-04-26

**Authors:** Emmanouil L. Papazoglou, Panagiotis Karmiris-Obratański, Beata Leszczyńska-Madej, Angelos P. Markopoulos

**Affiliations:** 1 School of Mechanical Engineering, Laboratory of Manufacturing Technology, National Technical University of Athens, Athens, Greece; 2 Department of Manufacturing Systems, Faculty of Mechanical Engineering and Robotics, AGH University of Science and Technology, Cracow, Poland; 3 Department of Materials Science and Non-Ferrous Metals Engineering, Faculty of Non-Ferrous Metals, AGH University of Science and Technology, Cracow, Poland

**Keywords:** Engineering, Materials science

## Abstract

Titanium alloys, due to their unique properties, are utilized in numerous modern high-end applications. Electrical Discharge Machining (EDM) is a non-conventional machining process, commonly used in machining of hard-to-cut materials. The current paper, presents an experimental study regarding the machining of Titanium Grade2 with EDM, coupled with the development of a simulation model. The machining performance indexes of Material Removal Rate, Tool Wear Ratio, and Average White Layer Thickness were measured and calculated for different pulse-on currents and pulse-on times. Moreover, the developed model that integrates a heat transfer analysis with deformed geometry, allows to estimate the power distribution between the electrode and the workpiece, as well as the Plasma Flushing Efficiency, giving an insight view of the process. Finally, by employing the Response Surface Methodology, educed regression models that correlate the machining parameters with the corresponding results, while for all the aforementioned indexes, ANOVA was performed.

## Introduction

Electrical Discharge Machining (EDM) is classified as one of the earliest non-conventional machining processes, but it still finds extensive use and application in modern industry as a leading edge machining process in treating hard-to-cut materials^1^. The fundamental principle of EDM is that material removal is resulted by means of rapid repetitive spark discharges, which occur between a working electrode and the workpiece. During EDM, a pulse voltage difference is applied between the workpiece and the electrode, both of which have to be immersed into proper dielectric fluid. Under specific conditions, i.e., voltage and gap between electrode and workpiece, an intense column of electromagnetic flux is formed, best known as plasma channel, having energy densities in the range of 10^11^–10^14^ W/m^2^. Due to the high electromagnetic energy density, extremely high temperatures are topically developed, i.e. 6000–12,000 °C, resulting in the melting and/or ablation of material from both the electrode and the workpiece^2,3^. EDM has been successfully used in the machining of hard-to-cut material, facilitating and achieving high dimensional accuracy, in complex shapes and geometries. Moreover, EDM is a non-contact machining process, since no contact exists between the working electrode and the workpiece, thus, no cutting forces are developed, leading to absence of mechanically induced residual stresses in the workpiece material. Due to the inherent advantages of EDM in comparison with conventional cutting processes and its capability to handle any electrical conductive material, regardless of its mechanical properties, EDM finds extensive use in the production of dies and molds, in automotive and aerospace industry, as well as in the field of surgical components production^4^.

EDM is a complicated, multi-parameter process; machining parameters include the pulse-on current (I_P_), the pulse-on time (T_on_), the duty factor (η), the machining voltage (V), the polarity, the discharge gap and the dielectric flushing pressure and method. At the same time, the machining results depend on the thermo-physical properties of the working electrode and the workpiece. Different materials like copper, graphite and tungsten have been proposed and tested as working electrodes, as well as composite and powder metallurgy-made materials^2,5^. The performance of the machining process is assessed mainly in terms of the Material Removal Rate (MRR), the Tool Wear Ratio (TWR), and the resulted machined Surface Quality (SQ). The improvement of MRR and the decrease of TWR are vital, in order for EDM to become economically competitive in comparison to conventional machining processes. Moreover, the decrease of TWR and the reduction of tool wear, affects beneficially the machining precision, as the electrode’s profile and geometry change at slower pace, keeping the machining precision at high level. Since EDM is a multi-parameter process, extended experiments have to be carried out so that the machining of a range of materials under different parameters can be studied. Thus, the development of models and simulations procure the advance of research, limiting the need of experiments, while, and at the same time, a more adequate and in-depth analysis of the process can be attained.

Modeling and simulation are not a straightforward and unambiguous process, since complicated multi-physical phenomena that occur, have to be modeled in an accurate and realistic way, considering the given limitations in computer processing power, as well as the lack of robust theoretical background. Definitely, there are some basic guidelines that are followed, nevertheless, there are certain topics concerning the modeling and simulation of EDM that are still scientifically open. More specifically, in the vast majority of EDM models, a single spark is simulated^6^, with conduction considered as the dominant heat transfer mechanism^7^. The MRR is estimated based on the developed temperature profile, as well as the resulted crater's geometrical characteristics, i.e. depth and width^8,9^. On the contrary, in many aspects, the proposed models differentiate as a result of a vague theoretical background. The plasma channel is simulated as a boundary heat flux, nevertheless, there are different approaches regarding its spatial and temporal distribution. In sake of simplicity, in some studies, the plasma is considered as a point heat source^10,11^, while in most cases, it is modeled either as a disk heat source or having a Gaussian spatial distribution^9,12^. Escobar et al.^13^ and Weingärtner et al.^14^ have presented interesting studies concerning the different simulation results that emerged by employing different types of heat sources in models. Adopting the more realistic approach that plasma channel has a spatial distribution, means that subsequently its radius has to be defined. Again, there is not a comprehensively accepted method, rather semi-empirical relations that correlate the plasma radius with the machining parameters I_P_ and T_on_^2,15^. In some simulations the plasma radius is considered as time dependent^16–18^, although there is no absolutely realistic way to confirm such models^19^. One more open research topic is the distribution of energy between the workpiece and electrode. In the most classical and simplified approach, according to DiBitonto et al.^10^, 18.3% of the plasma energy is absorbed by the workpiece, an estimation that has been adopted in several models^6,8,20^. On the other hand, experimental^21,22^ and simulation studies^7,15,16,23^ have proved that the absorbed by the workpiece amount of energy is not constant, but depends on the machining parameters, namely the pulse-on current and the pulse-on time. Klocke et al.^24^ have conducted an interesting study regarding the variability of existing models in simulation of EDM, concluding that different modeling parameters have a considerable discrepancy in obtained results. One more modeling aspect that necessitates careful definition is the Plasma Flushing Efficiency (PFE), which indicates the fraction of the molten material that is efficiently removed by the workpiece with every spark. Aiming on a simplified approach, in some simulations the PFE is considered 100%^25^ or silently neglected, implying that molten material is totally removed by the formatted crater. However, such a hypothesis is clearly inaccurate, since it cannot interpret the White Layer (WL) formation and be in agreement with experimental results. In more realistic approaches, the PFE is calculated in respect to machining parameters, according to experimental data, often using reverse engineering^9,23^. For all the aforementioned reasons, the importance of developing a model that is accurate, detailed and in agreement with experimental results, is deduced. It pertains to a model that not only explains and describes the occurring physical phenomena, but is also capable of predicting the results of the machining process.

Titanium-based alloys find extensive applicability in numerous modern industrial fields, such as aerospace, automobile, gas-turbine engines, nuclear and chemical industry, sports and medical applications. Their extended utilization is owned to their unique properties, namely the high strength at low to moderate temperature, the superior strength to weigh ratio, the excellent corrosion and wear resistance, the fatigue durability and their high biocompatibility^5,26^. Nevertheless, titanium alloys are considered hard-to-cut materials, suffering from poor machinability, due to their inherent mechanical and thermo-physical properties. Low thermal conductivity, high chemical reactivity, as well as the low modulus of elasticity are the main drawbacks in their machining with conventional processes^27,28^. Thus, non-conventional machining techniques, like EDM, are utilized as feasible alternatives.

Already from the late 90' s the machining of titanium alloys with EDM gathered the research interest, with experimental studies being conducted mainly. The MRR, TWR and SQ were the most common research topics, and the way that these machining performance indexes are effected by the machining conditions, i.e. the pulse-on current, pulse-on time, the dielectric fluid, and the electrode material^29–31^. The significantly low MRR and high TWR lead to the use of different electrode materials, namely graphite, copper, aluminum or composite, in order to render the process more efficient and thus, economically feasible^32–34^. As Fonda et al.^1^ concluded, the productivity in machining Ti–6A–4V (the most commonly used titanium alloy) with EDM, depends directly to the temperature of the workpiece during machining, entailing that the properly chosen machining parameters are of great importance. Moreover, the energy distribution between electrode and workpiece is a fundamental cause of the low MRR and the high TWR that is observed in machining titanium alloys with EDM. Shen et al.^35^ investigated this distribution, inferring that the energy distribution ratio is highly affected by the machining parameters, namely, the energy density, the pulse duration and the polarity. Some peculiarities in machining of titanium based alloys with EDM have been investigated by Sen et al.^36^ and Holsten et al.^37^. The former studied the significant change in machinability of Ti–6Al–4V with the addition of Boron, and as the Boron content and the grain size change. The latter investigators, in a conceptually similar study, ascertained the differences in machining titanium alloys according to their Al content, emphasizing on the major role of polarity and dielectric fluid type. Although many researches have been carried out concerning the machining of Ti–6Al–4V and Ti–6Al–4V ELI with EDM^38–41^, research regarding other titanium grades is limited and probably inadequate^42–44^. Ahuja et al.^45^ presented a study regarding the bioactivity of commercially pure titanium processed by micro-electric discharge drilling, establishing that EDM is a feasible method for titanium machining. Finally, and under the scope of utilizing titanium alloys in bio-medical applications, the Ti-6Al-4Nb (an alloy where the cytotoxic vanadium has been replaced by niobium) was studied, concerning its surface integrity, its bio-activity and the performance characteristics during and after its machining with EDM^46^.

**Figure 1:**
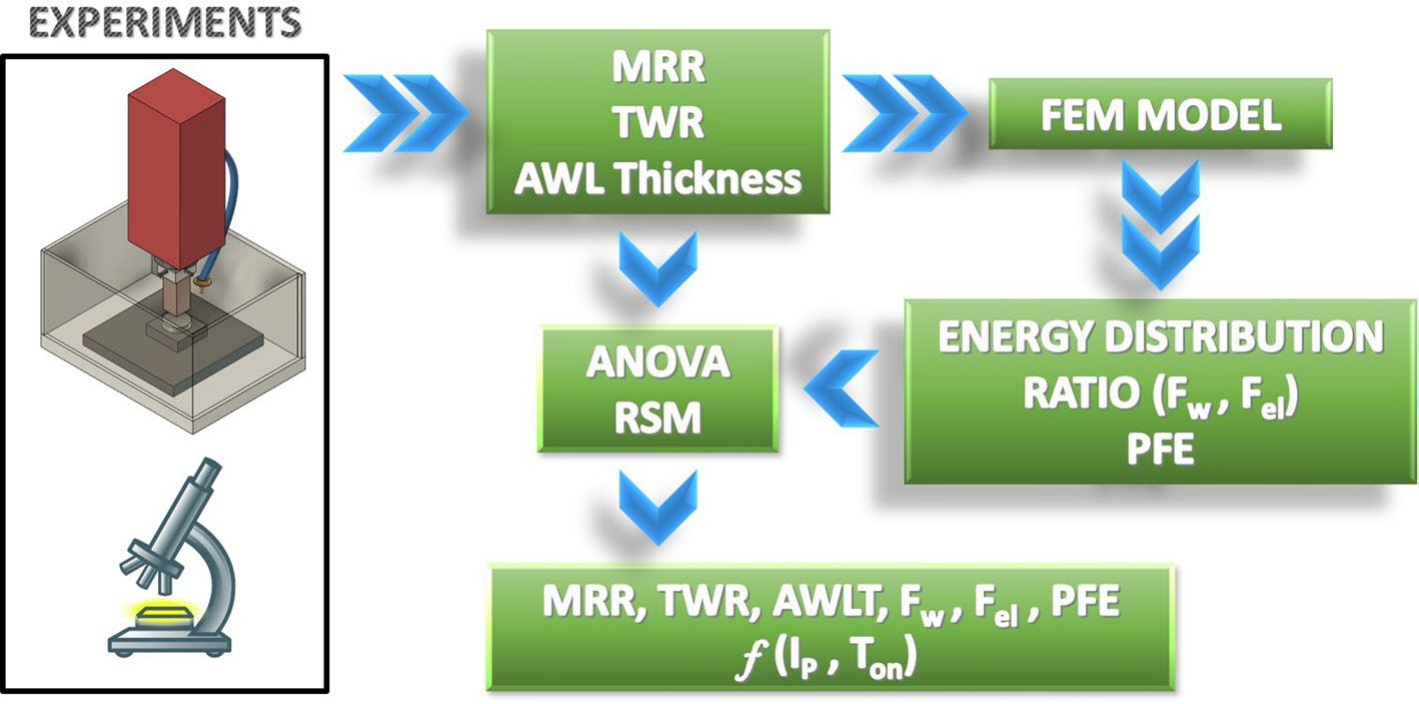
Graphical overview.

The objective of the current study is to present a thorough research regarding the machining of pure titanium with EDM. The main performance indexes will be measured and evaluated, while, at the same time, and by employing simulation models, parameters and coefficients that are extremely difficult, or even impossible to be experimentally measured, will be estimated. At first, a series of experiments was conducted, in machining Titanium Grade2 with EDM, by using graphite electrode, and for a wide range of per pulse energy, i.e., from 6.75 up to 150 mJ. The MRR and the TWR were calculated, and by metallographic analysis the Average White Layer Thickness (AWLT) was measured. Subsequently, to simulate the process, a detailed Finite Element Method (FEM) model was developed, integrating a heat transfer analysis with deformed geometry, aiming to more realistic and accurate results. Employing the experimental data and through reverse engineering, important parameters of the process, such as the energy distribution ratio between the workpiece and the electrode, the PFE, as well as secondary data like the craters' geometrical characteristics, were determined. Finally, based on Response Surface Methodology (RSM), an Analysis of Variance (ANOVA) was performed, while equations that correlate the machining parameters with the most important process' performance indexes were proposed. In summation, the aim of the current paper is to present a comprehensive investigation of machining Titanium Grade2 with EDM, providing experimental, and simulation data, while via the semi-empirical correlations, a predictability, regarding the process, is gained. The aim of the current study is to provide scientifically interesting results, which will advance and promote further research in the relevant field, along with useful and more applicable data. In Fig. 1 the flow chart presents a graphical overview of the current study.

## Experimental procedure

The experiments were conducted in an Agie-Charmilles Roboform 350 Sp Die-Sinking EDM employing square type of pulses. A plate of Titanium Grade2 (see Table 1 for Titanium Grade2 chemical composition and the maximum accepted limits of elements) was used as a workpiece, and a graphite working electrode, with nominal dimensions of 19 × 14 mm. Synthetic hydrocarbon oil (kerosene) was utilized as dielectric fluid, which was properly channeled into the working tank through a flushing nozzle. A full-scale experiment was carried out, with 4-levels control parameters the pulse-on current and the pulse-on time, taking values from 9 up to 25A and 25 up to 200 μs, respectively. That way, a wide range of per pulse energies was studied, i.e. 6.75 mJ up to 150 mJ, giving a justified reliability on the subsequently developed models and the conclusions that were drawn. All other machining parameters were kept constant, namely, the duty factor was set at 50%, the flushing pressure at 0.7MP and the nominal cutting depth at 0.5 mm. In Table 2 the machining parameters are listed in detail.

**Table 1:** Titanium Grade2 chemical composition.

Ti	Max				
	Fe	O	C	N	H
Bal (%)	0.3	0.25	0.1	0.03	0.015

**Table 2:** Experimental parameters.

Machining conditions	Level 1	Level 2	Level 3	Level 4
Discharge current I_p_ [A]	9	13	17	25
Pulse on-time T_on_ [μs]	25	50	100	200
Duty Factor	0.5			
Close circuit voltage [V]	30			
Dielectric	Synthetic hydrocarbon fluid			
Dielectric flushing	Side flushing with pressure			
Dielectric flushing pressure [MPa]	0.7 (constant under whole conditions)			

The MRR and the TWR were calculated according to Eqs. (1) and (2) respectively^47^.

1 $$MRR = \frac{{W_{st} - W_{fin} }}{{t_{mach} }} \cdot \frac{1}{\rho }$$

2 $$TWR = \frac{{El_{st} - El_{fin} }}{{W_{st} - W_{fin} }}$$

where MRR in [mm^3^/min], W_st_, W_fin_ the workpiece weight before and after the machining respectively in [gr], t_mach_ the machining time in [min], ρ the workpiece material density in [gr/mm^3^], and El_st_, El_fin_ the working electrode weight before and after machining respectively in [gr]. It has to be pointed out that in-between experiments the electrode was properly dried, to remove any absorbed dielectric, since graphite is, by nature, a porous medium.

**Figure 2:**
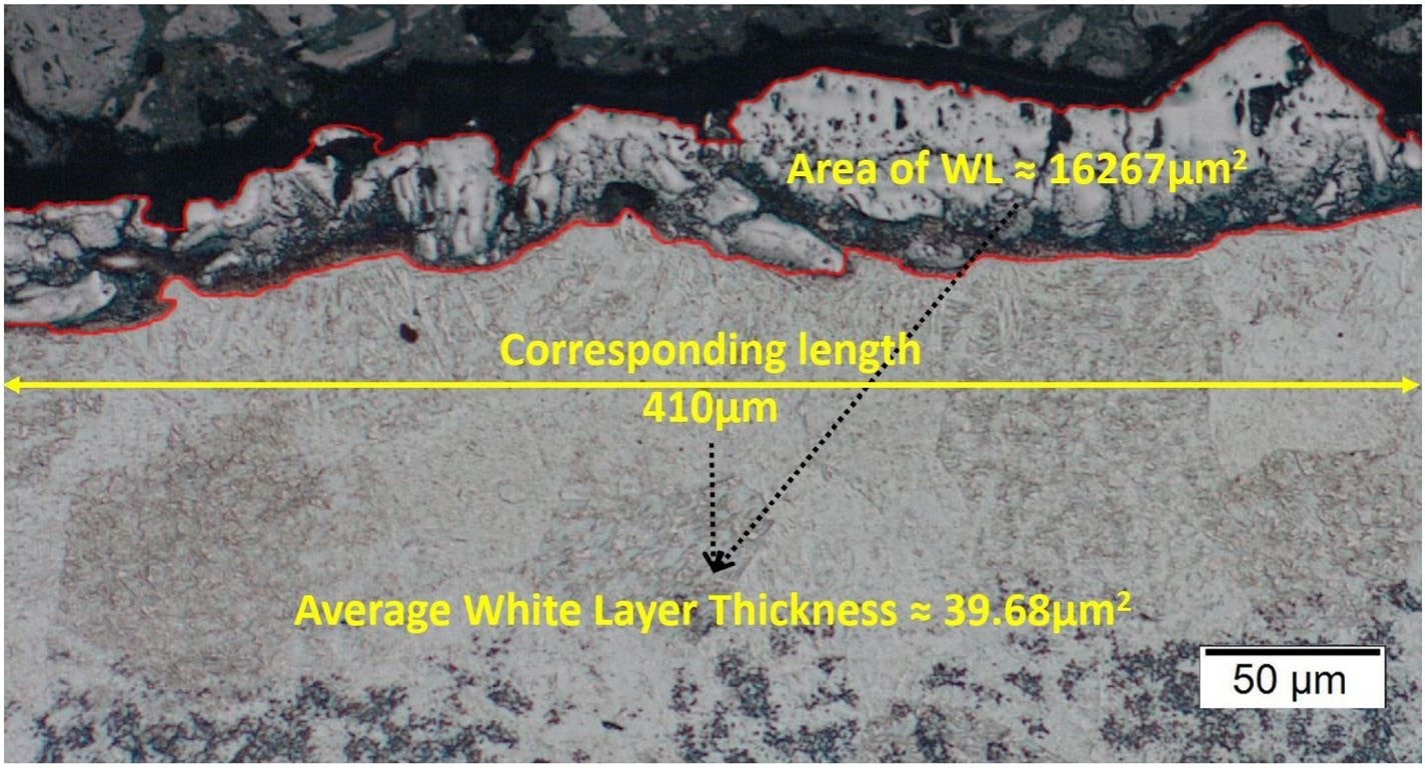
Measurement of the AWLT for I_P_ 25A and T_on_ 200 μs.

Following the machining, a metallographic analysis of the cross-section of the workpiece took place. The specimens were cut perpendicular to the machined surfaces and after the necessary chemical etching, they were observed in optical microscope in order for the AWLT to be estimated. The AWLT was calculated as the quotient of the WL area divided by the corresponding length. In Fig. 2 an enlightening snapshot of the measuring method is depicted.

## Modeling and simulation

In the current study, a FEM model is developed and proposed in order to simulate the material erosion during EDM. Since EDM is by nature a stochastic and chaotic process, some basic assumptions and simplifications have to be made, in order for its modeling and simulation to become attainable.

In the current model, the following assumption/simplifications have been considered:

- At first, the concept of a "typical spark" is introduced, implying that all the sparks are identical, and a single plasma channel is formed on each discharge. In the work of Klocke et al.^24^ is stated that the single discharge experiments do not represent the material removal of a continuous discharge process. Hence, the introduction of a "typical spark" that can describe the continuous process is preferable than conducting and modeling single discharge experiments.
- Conduction is regarded as the main and only heat transfer mechanism into the material^48^.
- The energy distribution between workpiece and electrode depends on the machining parameters.
- The plasma channel is modeled employing a Gaussian heat flux distribution^17,23^.
- The erosion of the molten material is taken into consideration, while the PFE also depends on the machining parameters.
- The material is considered homogenous and isotropic, with temperature dependent properties. Moreover, latent heats of the materials are also considered.
- As only a relevantly small material volume is removed per spark, compared to workpiece and tool electrodes’ volumes, both the workpiece and the electrode can be considered as semi-infinite bodies.

### Governing equations and boundary conditions

The conduction heat transfer, the main and only mechanism of heat transfer into the material, is mathematically expressed by Eq. (3)^12,49^:

3 $$\left. {\begin{array}{*{20}c} {\rho \cdot Cp \cdot \frac{\partial T}{{\partial t}} + \nabla {\text{q}} = Q} \\ {{\text{q}} = - k \cdot \nabla T} \\ \end{array} } \right\} \Rightarrow \rho \cdot Cp \cdot \frac{\partial T}{{\partial t}} - \nabla \left( {k \cdot \nabla T} \right) = Q$$

with T the temperature in [K], ρ the density in [kg/m^3^], Cp the heat capacity in [J/kgK], k the thermal conductivity in [W/mK], and Q a heat source or a heat sink in [W/m^3^].

Since the plasma channel is modeled as a heat flux with Gaussian distribution, it can be described as^12,49^:

4 $$q_{pl} \left( r \right) = q_{o} \exp \left( { - \frac{{r^{2} }}{{2\sigma^{2} }}} \right)$$

with q_pl_ being the heat flux due to the plasma channel in [W/m^2^], Q_o_ the maximum power intensity at the center of the plasma channel in [W/m^2^], r the distance from the center of the plasma channel in [m], and σ the standard deviation of the Gaussian distribution. Considering that the plasma channel radius R_pl_ [m] is equal to three standard deviations (R_pl_ = 3σ), and that the integral of the Gaussian distribution has to be coequal with the total absorbed power, the Q_o_ can be calculated according to Eq. (5)^12,49^:

5 $$\oint {q(r)dA = F_{i} \cdot V \cdot I_{P} \Rightarrow \int\limits_{0}^{{R_{pl} }} {q(r) \cdot 2\pi rdr = } } F_{i} \cdot V \cdot I_{P} \Rightarrow Q_{o} = 4.57\frac{{F_{i} VI_{P} }}{{\pi R_{pl}^{2} }}$$

with F_i_ being the fraction of energy that is absorbed either by the workpiece (F_w_) or by the electrode (F_el_), V the voltage in [V], and I_P_ the pulse-on current in [A]. Since square pulses were employed, the pulse-on current and the voltage are considered steady throughout the discharge time. Hence, the power distribution can be expressed as^12,49^:

6 $$q_{pl} \left( r \right) = 4.57\frac{{F_{i} VI_{P} }}{{\pi R_{pl}^{2} }}\exp \left( { - 4.5\left( {\frac{r}{{R_{pl} }}} \right)^{2} } \right)$$

It was previously mentioned that there is a divergence of views regarding the method of estimation of the plasma radius, since no adequately robust theoretical model exists, but only semi-empirical relations. In a previous study^49^ it has been determined that when a thermal model is coupled with deformed geometry, the most suited correlation between machining parameters and plasma radius, leading in more accurate and realistic results is^2,49^:

7 $$R_{pl} = 0.85 \cdot 10^{ - 3} \cdot I_{P}^{0.48} \cdot T_{on}^{0.35}$$

with R_pl_ the plasma radius in [m].

As both electrode and workpiece are submerged into the dielectric fluid, heat flux due to convection takes place, which is calculated as^50^:

8 $$q_{conv} = h_{diel} \left( {T - T_{diel} } \right)$$

with q_conv_ being the heat flux from the workpiece to dielectric fluid due to convection in [W/m^2^], h_diel_ the heat transfer coefficient between the workpiece and the dielectric oil with value h_diel_ = 10^5^ [W/(m^2^K)]^51^ and T_diel_ the dielectric oil temperature and equal to 293.15 K.

The heat losses due to radiation can be estimated as^50^:

9 $$q_{rad} = \varepsilon_{i} \left( {T^{4} - T_{amb}^{4} } \right)$$

with q_rad_ being the heat flux due to radiation in [W/m^2^], ε_i_ the emissivity coefficient of the electrode (ε_el_) or the workpiece (ε_w_), T_amb_ the ambient temperature in [K].

As it was previously stated, the current model couples heat transfer analysis with deformed geometry that simulates the material erosion. The material erosion denotes the simultaneous energy removal from the system; hence, the erosion rate can be calculated in respect to the removed energy rate. Assuming that the total amount of molten (in case of the graphite electrode, ablated) material is removed, the surface temperature remains constant just below the phase change temperature, the removed energy rate can be calculated by employing Eq. (10)^49^:

10 $$q_{ph.ch.} = h_{ph.ch.} \left( {T_{ph.ch.} - T} \right)\;with\;h_{ph.ch.} \left\{ {\begin{array}{*{20}l} 0 \hfill & {for} \hfill & {T \le T_{ph.ch.} } \hfill \\ {10^{9} } \hfill & {for} \hfill & {T > T_{ph.ch.} } \hfill \\ \end{array} } \right.$$

with q_ph.ch._ being the heat flux due to material melting in [W/m^2^], T_ph.ch._ the material’s phase change temperature in [K] and h_ph.ch._ the heat transfer coefficient in [W/(m^2^K)], which is zero for T ≤ T_ph.ch._ and increases linearly for T > T_ph.ch._.

In basis of Eq. (10), the erosion rate, i.e. the normal velocity of the eroding surface, can be defined as^49^:

11 $$u_{ph.ch.} = \frac{{q_{melt} }}{{\rho_{i} \left( {Cp\left( {T - T_{ph.ch.} } \right) + LH_{i} } \right)}}$$

with u_ph.ch._ being the material’s eroding velocity in [m/s], ρ_i_ the density of the electrode or the workpiece in [kg/m^3^], and LH_i_ the material’s latent heat in [J/kg]. In case of the Titanium workpiece the latent heat of phase change refers to the latent heat of melting, while for the graphite electrode to the latent heat of ablation.

At this point, the model's boundary conditions have been fully defined, except of the absorption coefficients F_w_ and F_el_, which are calculated through reverse engineering. Conceptually, reverse engineering is a method to estimate an unknown parameter, which cannot be defined experimentally or analytically, by tuning it in such a way that simulation is in line with experimental results. In the current model the inputs parameters, based on which the absorption coefficients were tuned/calculated are the "average crater volume” (ACV) (WACV and EACV for workpiece and electrode, respectively) and the AWLT. The WACV and EACV are directly resulted by the MRR and TWR, while the AWLT was measured through metallographic analysis, see Experimental Procedure section.

In workpiece simulation, at some point during the spark-on time, when the formatted crater's volume becomes equal to WACV, the material removal is adjusted to zero (by zeroing the q_melt_), thus, overheated volume of material (T > T_melt_) is formed. This melted, but not abstracted material, forms the WL, which, for a certain value of F_w_, has thickness equal to AWLT. On the other hand, graphite does not form any type of WL since for relatively low pressures it does not melt, but sublimes, for temperature near 4000 K. Consequently, the electrode absorption coefficient is calculated based on the EACV, namely, for the F_el_ that the formatted crater's volume equals to EACV. The WACV and EACV can be calculated by Eqs. (12) and (13) respectively.

12 $$WACV = 10^{ - 9} \left( {\frac{MRR}{{number\,of\, sparks\,per\,\min }}} \right) = 10^{ - 9} \left( {\frac{MRR}{{\eta \left( {\frac{60}{{T_{on} \cdot 10^{ - 6} }}} \right)}}} \right)$$

13 $$EACV = WACV \cdot TWR \cdot \frac{{\rho_{gr} }}{{\rho_{ti} }}$$

with WACV and EACV being the workpiece and electrode average crater volumes, respectively in [m^3^], T_on_ the pulse-on time in [μs], ρ_gr_, ρ_ti_ the densities of graphite and Titanium Grade2, respectively in [kg/m^3^], and η the duty factor.

Finally, the PFE can be estimated. To calculate the PFE, maximum theoretical crater volume has to be defined, namely, the crater volume in case of a zero WL. Having already defined the workpiece absorption coefficient, the maximum theoretical average crater volume (WACV_theor_) is computed by allowing the material removal through the whole pulse-on time, hence, no volume of not abstracted melt, i.e., WL, is formed. Knowing the WACV_theor_, PFE can be estimated according to Eq. (14)^52^:

14 $$PFE = \frac{WACV}{{WACV_{theor} }}$$

**Figure 3:**
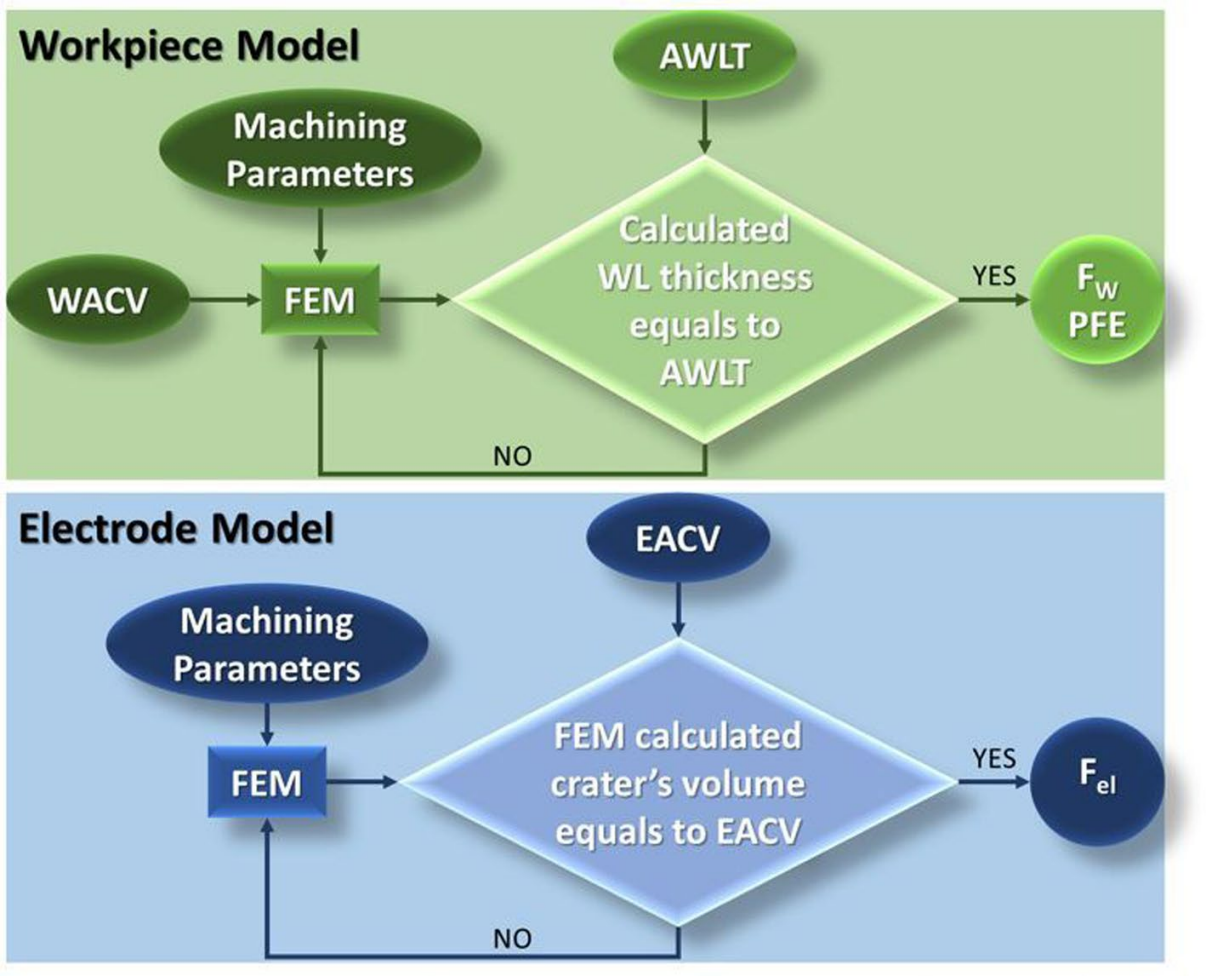
Flow chart of the reverse engineering method.

In Fig. 3, by a flow chart, the reverse engineering method that was analyzed afore is graphically presented.

### Material properties and material models

Weingärtner et al.^14^ in their study indicated the significance of a comprehensive and accurate material's thermophysical properties definition. In the current study, the major materials’ properties and constants were determined as temperature dependent according to literature, while the phase change latent heats were also taken into consideration, since they are in the core of the model.

For Titanium Grade2, the thermo-physical properties values, as Mills^53^ recommends them, were adopted. Density, specific heat, thermal conductivity and emissivity were defined and introduced into the model, as temperature dependent. Moreover, the specific heat of phase change between Titanium phase α and Titanium phase β were also encompassed. The transition from phase α to phase β is temperature defined at 1155 [K] with latent heat of transition LH_α→β_ = 87 [kJ/kg], while the melting point of Titanium Grade2 set on 1941 [K] with melting latent heat LH_melt_ = 295[kJ/kg].

The modeling of electrode material is more complicated, since the graphite electrode is a porous medium, and thus, the thermo-physical properties have to be defined properly, considering its porosity. The effective volumetric heat capacity is calculated according to Eq. (15)^54^ :

15 $$\left( {\rho Cp} \right)_{eff} = \phi_{gr} \rho_{gr} Cp_{gr} + \left( {1 - \phi_{gr} } \right)\rho_{\ker } Cp_{\ker }$$

with φ_gra_ being the volume fraction of graphite, ρ_gr_ and ρ_ker_ [kg/m^3^] the densities of graphite and kerosene respectively and Cp_gr_ and Cp_ker_ the specific heats in [J/kgK]. According to the supplier, the electrode has a nominal density of 1.8 [g/cm^3^], while graphite's density is 2.26 [g/cm^3^]^55^, thus, the graphite volume fraction in the electrode can be estimated as φ_gra_≈1.8/2.26≈0.796. On the contrary with the calculation of the effective volumetric heat capacity, where the volume average method was employed, the effective thermal conductivity has to be defined based on the power law^54^:

16 $$k_{eff} = k_{gr}^{{\phi_{gr} }} \cdot k_{\ker }^{{\left( {1 - \phi_{gr} } \right)}}$$

with k_eff_ being the effective thermal conductivity in [W/mK], and k_gr_ and k_ker_ the thermal conductivity of graphite and kerosene, respectively, in [W/mK]. Since graphite is an anisotropic material, its relative thermal conductivity is used, based on values that are reported by Pierson^55^. For the graphite thermo-physical properties, the values that are referred by Pierson^55^ were used, while for kerosene the ones cited by Zhang et al.^56^ are employed. As in titanium, the phase change latent heats were also were considered and integrated into the model. The latent heat of sublimation for graphite defined as LH_sub_ = 39,716 [kJ/kg] at 4000 [K], while for the kerosene the ablation latent heat LH_abl_ = 251 [kJ/kg] at 502 [K].

### Modeling parameters

**Figure 4:**
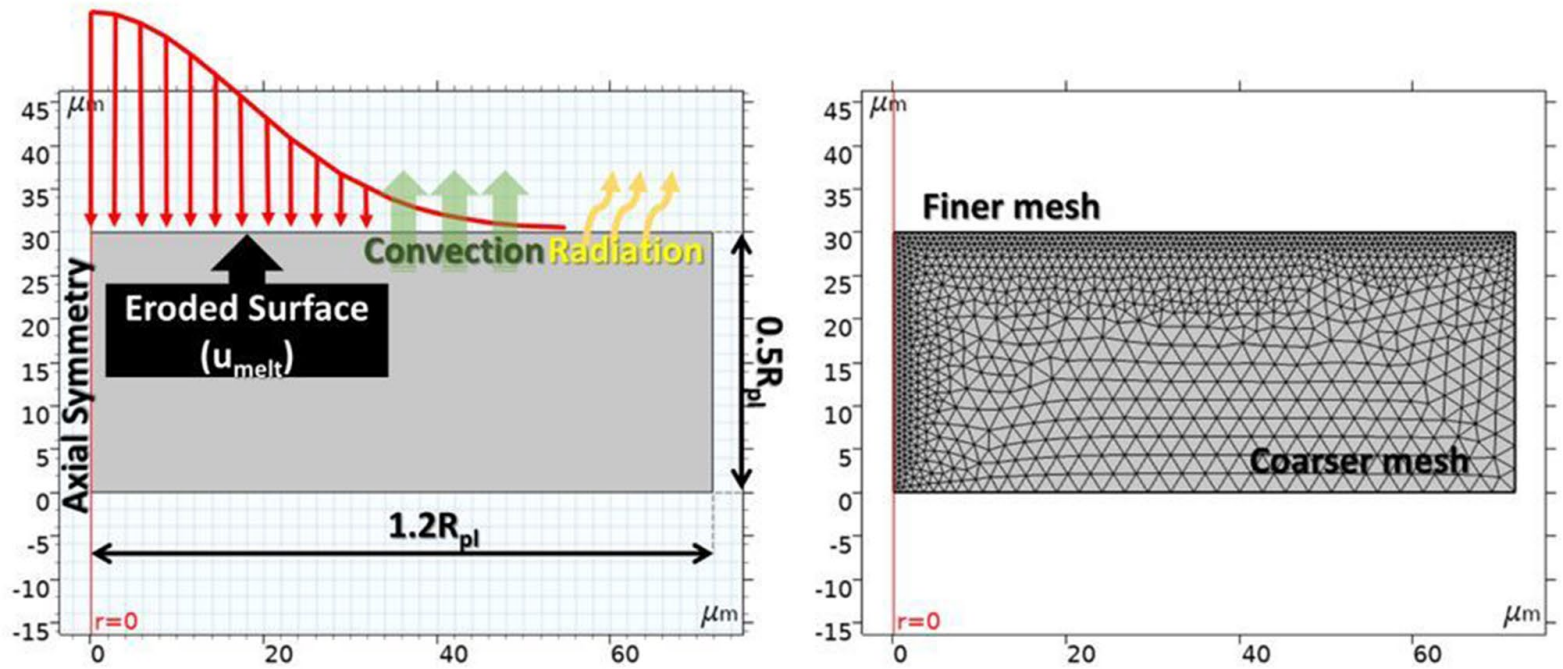
Model’s boundary conditions and meshing.

Aiming at economy in computational time and power, the model control volume was set up as axonometric, a justified selection since the spark is considered as uniform. The control volume dimension was defined in respect to the plasma radius; specifically, the width was set equal to 1.2 of the R_pl_ and the height to 0.5 of the R_pl_. Moreover, a scaled mesh was adopted, finer in the volume where high gradients of temperature and material erosion occur and coarser in the remaining volume, see Fig. 4, while mesh independence was ensured. Finally, the maximum time step was set at 0.1 μs. In Fig. 4 the boundary conditions, as well as the mesh of the control volume are presented.

### Response surface methodology

Response Surface Methodology (RSM) is a collection of mathematical and statistical techniques, which are generally used in modeling and analysis of a problem, namely, to determine the correlation between several explanatory variables and response variables. The next step after the Design of Experiment (DOE), is to define the form of relationship between response and independent variables. Optimum model may consist of linear, squared and cross-product terms of the independent variables. Model's coefficients are estimated based on the least squares method, while, subsequently, ANOVA is commonly performed to define the adequacy of the model and the statistical importance of its terms. There is no general rule regarding the order of model that is best suited on each problem, rather than a trial-and-error procedure, driven by the obtained results. The RSM can be mathematically expressed as^57,58^:

17 $$f\left( {x_{1} ,x_{2} , \ldots ,x_{k} } \right) = a_{o} + \underbrace {{\sum\limits_{i = 1}^{k} {a_{i} x_{i} } }}_{{linear\,terms}} + \underbrace {{\sum\limits_{i = 1}^{k} {a_{ii} x_{i}^{2} } }}_{{squared\,terms}} + \underbrace {{\mathop {\sum {\sum {a_{ij} } } x_{i} x_{j} }\limits_{i < j} }}_{{cross - product\,terms}}$$

with f(x_1_,x_2_,…,x_k_) being the corresponding response variable yield. In studying and modeling the EDM process, RSM is frequently employed^43,59,60^.

## Results and discussion

### Material removal rate, tool wear ratio and average white layer thickness

In Table 3 the experimental results regarding the MRR, TWR, and AWLT are presented.

**Table 3:** Experimental results.

#	I_p_ [A]	T_on_ [μs]	MRR	TWR	AWLT [μm]
1	9	25	0.2782	0.911	5.71
2	9	50	0.2944	0.617	10.60
3	9	100	0.2763	0.829	11.94
4	9	200	0.2134	0.569	21.84
5	13	25	0.6382	1.017	10.24
6	13	50	0.7214	0.959	10.16
7	13	100	0.6176	0.802	17.22
8	13	200	0.5440	0.699	28.92
9	17	25	0.6804	1.028	12.70
10	17	50	0.8749	0.838	9.33
11	17	100	0.8498	0.777	16.83
12	17	200	0.8235	0.668	28.47
13	25	25	0.8589	1.037	13.89
14	25	50	1.2486	0.810	15.20
15	25	100	1.4208	0.708	21.54
16	25	200	1.5505	0.535	39.68

**Figure 5:**
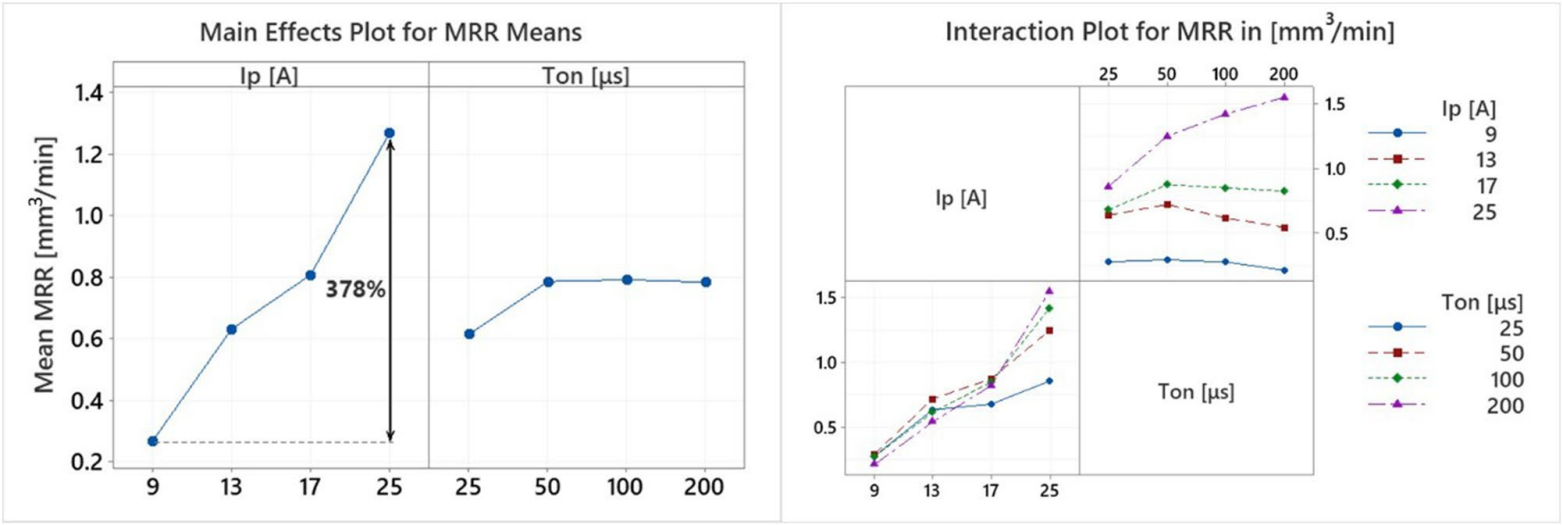
Main Effects Plot and Interaction Plot of MRR.

MRR and TWR are major productivity and efficiency indexes, straight related with machining parameters, and affected by the machining power and the per pulse discharge energy. EDM is a multi-parameter complicated process, with a nonlinear response, indicating that increase in machining power, or in per pulse discharge energy, does not compulsorily leads in increased MRR. On the contrary, there is a limit to the maximum attained MRR, and by employing more intense machining conditions, e.g. increased per pulse energy, MRR not only may not increase, but it could be negatively affected. This MRR limit can be attributed to three main reasons: the plasma growth, the carbon decomposition and its subsequent deposition, and the debris concentration. Increased pulse duration leads to an expanding plasma channel that consumes a significant amount of energy. At the same time, high localized temperatures, due to increased pulse-on time, result to the decomposition of dielectric fluid's carbon, which subsequently is deposited and bonded on the electrode and the workpiece. This carbon and carbide layer, acts like a "barrier" and "shield", decreasing the machining efficiency and the MRR, while on the other hand, affects beneficially the electrode by shielding it and limiting its wear. Likewise, the debris that concentrate between the electrode and the workpiece act as a "barrier" as well. More intense machining conditions and/or extended pulse duration, increase their concentration, resulting to an amount of energy to be consumed by their re-melting. The in-brief aforementioned mechanisms that take place during machining indicate the complexity of the process, as well as the necessity of its comprehensive and thorough study. In Fig. 5, the Main Effects Plot and the Interaction Plot of MRR are presented.

Obviously, the MRR behavior confirms the above discussed underlying mechanisms. The pulse-on current has a clear effect on MRR, as increase of I_p_ results to an increase in MRR; as the interaction plot indicates, this tendency applies to all pulse-on times. Specifically, the mean MRR rises by 378% as the I_p_ increases from 9 to 25A. On the other hand, T_on_ affects in an ambiguous way the MRR, since, up to 50 μs pulse duration, an increase in MRR is recorded, while, for higher pulse-on times MRR is mainly stable or slightly decreased. Only for I_p_ 25A the MRR keeps increasing for higher T_on_. These conclusions are in line with the relevant literature; Chen et al.^29^ and Kumar et al.^39^ have reported similar results concerning the EDM of Ti–6Al–4V, namely, the increase of T_on_ beyond a certain limit is indifferently or even negatively affecting MRR. Taking in mind the obtained results, it would be justified to say that, as a rule of thumb, a higher MRR could easier be obtained by increasing the machining power, i.e., the pulse-on current, rather than by utilizing higher pulse-on times. On the other hand, these observations concern only MRR and no other machining results, namely TWR, AWLT or SQ, thus, a higher I_p_ is not always an appropriate solution.

An exceedingly useful tool in production planning is the capability of accurately predicting the machining results in respect to the machining parameters. Thus, employing the RSM, a correlation model between pulse-on current, pulse-on time and MRR is proposed:

18 $$MRR = - 0.323 + 0.0745I_{P} + 0.00017T_{on} - 0.001095I_{p}^{2} - 0.000016T_{on}^{2} + 0.000255I_{P} T_{on}$$

with MRR in [mm^3^/min], I_p_ in [A], and T_on_ in [μs].

**Figure 6:**
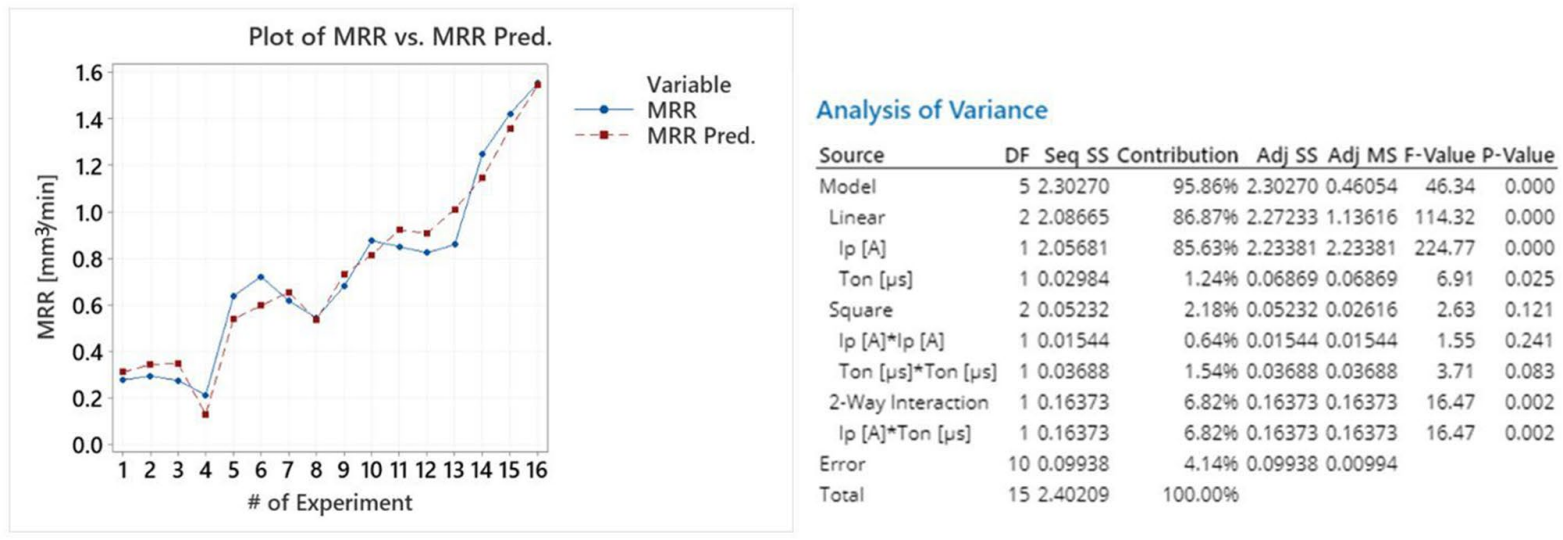
Plot of MRR vs MRR predicted and the model’s ANOVA.

The proposed model has a high level of fit, with R-sq greater than 95% and S value lower than 0.1. Moreover, the adequate fit is also confirmed by the p-value of the model, see Fig. 6, which is less than 0.05. At this point, it is interesting to highlight that I_p_ and the interaction of I_p_ with T_on_ have the highest contribution in MRR, implying that pulse-on current, and not pulse-on time, mainly affects the MRR, a conclusion that is in absolute agreement with experimental results. The model's appropriate fit that was theoretically analyzed above, is depicted in Fig. 6 where the MRR and the MRR Predicted values are plotted, having only a slight deviation.

**Figure 7:**
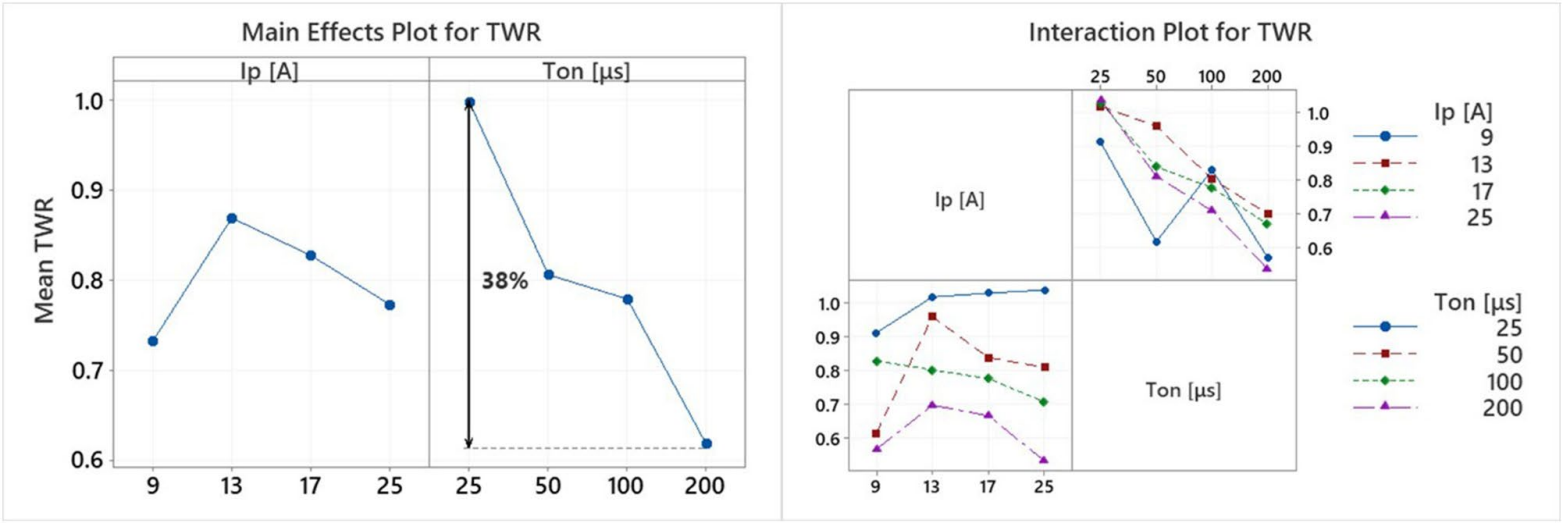
Main Effects Plot and Interaction Plot of TWR.

TWR is the other major index for evaluating the efficiency and the feasibility of the process. Based on the theory and the previously discussed, TWR is expected to respond differently than MRR in respect to machining parameters variation. In Fig. 7, the Main Effects Plot and the Interaction Plot for TWR are presented. The pulse-on current seems to have a fuzzy effect on TWR, since for 13A, a maximum is observed, while for further increase in I_p_, the mean TWR decreases. By a more in-depth analysis, and considering the interaction plot, it is concluded that indeed for 13A I_p_ the highest TWR is recorded, while the lowest one, depending on the pulse-on time, is defined at 9 or 25A. On the other hand, T_on_ affects the TWR in a clear manner. Namely, the increase of pulse-on time results to a consecutive decrease in TWR. Finally, the interaction plot indicates that only for I_p_ of 9A, TWR has a vague response to the increase of T_on_. Kumar et al.^42^ have presented analogous results and a similar interpretation regarding the shielding effect of decomposed carbon as well.

As in MRR, the prediction competence of TWR is extremely useful and advantageous in planning a feasible production. Hence, employing the RSM, a relation between machining parameters and TWR can be obtained:

19 $$TWR = 0.529 + 0.0615I_{P} - 0.00291T_{on} - 0.00158I_{p}^{2} + 0.000009T_{on}^{2} - 0.00007I_{P} T_{on}$$

with I_p_ in [A], and T_on_ in [μs].

**Figure 8:**
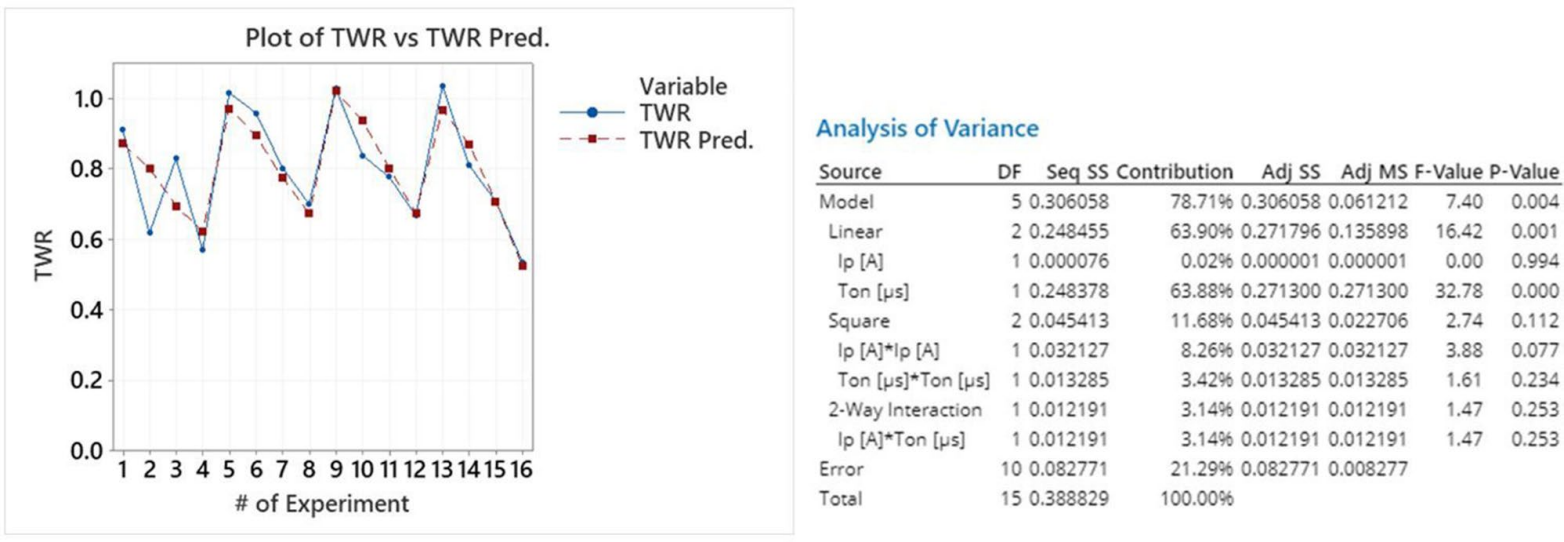
Plot of TWR vs TWR predicted and the model’s ANOVA.

The fit of the regression is considered sufficient, with R-sq 78.71%, S value less than 0.1, and model's P value 0.004, entailing that model can explain variations in the response. Comparatively with MRR, for TWR the parameter of major importance is the pulse-on time, having a 63.88% contribution, see Fig. 8, to the model's total Sequential Sums of Squares (Seq SS). Finally, by plotting the TWR values along with the model predicted ones, see Fig. 8, its suitability is confirmed, as it can follow the change of TWR in respect to machining parameters.

**Figure 9:**
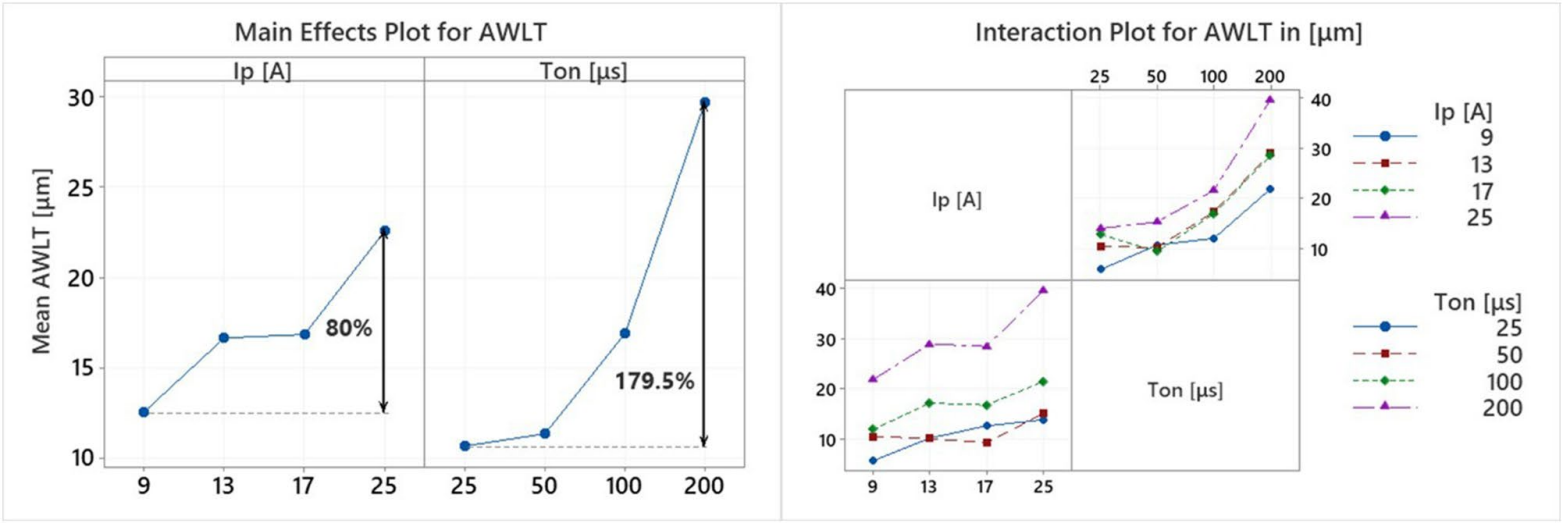
Main Effects Plot and Interaction Plot of AWLT.

Closing the current section, the formation of the WL will be discussed. During EDM, only an amount of molten material is efficiently removed by the workpiece, with the rest of it being re-solidified. Additionally, debris and/or ablated material that have remained close to the surface, may be re-condensed and adhered on the surface. The re-solidified and re-condensed material forms a layer known as White Layer. WL consist of amorphous material since it was solidified under extremely high cooling rates, thus it is easily distinct after the proper chemical etching. AWLT is an important parameter in EDM since it is related with the SQ, cracks formation and part's mechanical properties. Izman et al.^61^ have pointed out that lower WL thickness reduces the risk of part premature failure during operation, while Mower^62^ proved a reduction of fatigue strength, attributed to the presence of stress concentrating defects within the EDM recast layers. Thus, the AWLT is an important parameter in the production planning, as it is associated with manufactured component’s functionality. Figure 9 presents the change in AWLT depending on the machining parameters.

As the Main Effects Plot and Interaction Plot of Fig. 9 indicate, the AWLT is clearly affected by pulse-on current and time. Increase of I_p_ and T_on_ results to higher AWLT. More specifically, as the machining current increased from 9 to 25A the AWLT was increased 80%, while an increase in T_on_ from 25 to 200 μs resulted to a 179% thicker WL. The Interaction Plot totally confirms this interaction, since, in most cases, for more intense machining parameters, the AWLT increases. The rapid increase of the AWLT for higher pulse-on times consists a reasonable explanation regarding the reason behind the fact that for higher T_on_s the mean MRR does not keep increasing, but on the contrary, it is stabilized. Although a greater material volume melts, it is not be removed, but it is re-solidified forming a thicker WL. Hence, with an increase to pulse-on time, the mean MRR does not have a significant increase for T_on_s greater than 50 μs, while the mean AWLT is constantly increased.

**Figure 10:**
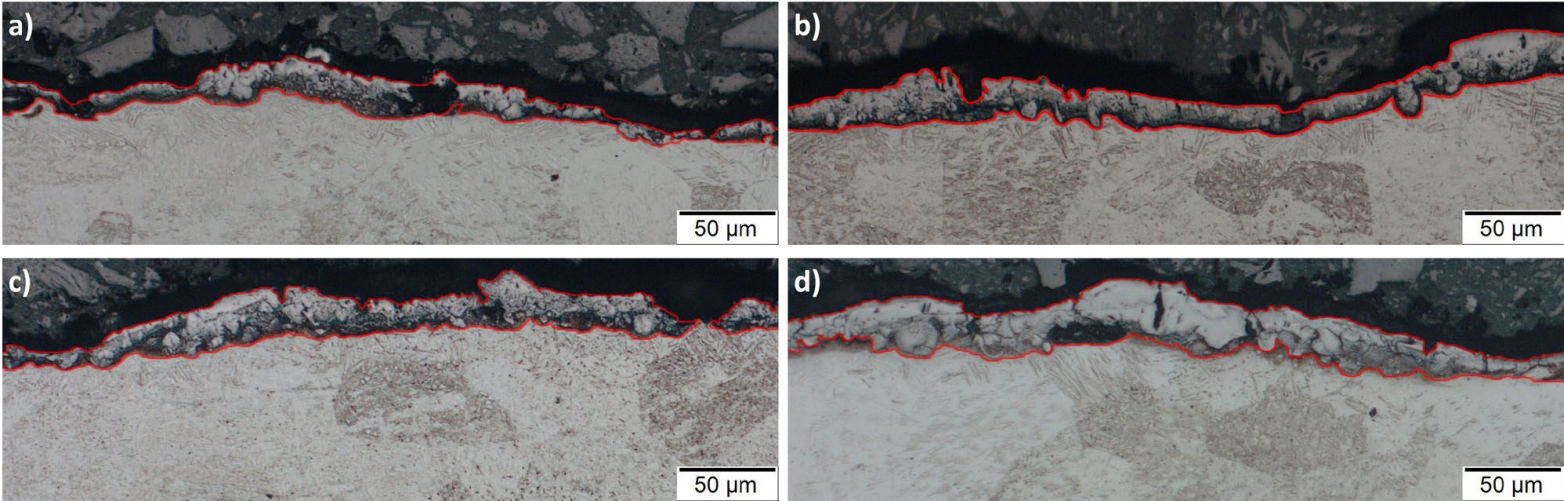
The WL for pulse-on current 17A and pulse-on times (**a**) 25 μs, (**b**) 50 μs, (**c**) 100 μs and (**d**) 200 μs.

The main causes of WL increased thickness for more intense pulse-on currents and times are the increase in machining power and per-pulse energy, as well the increasing inefficiency of the dielectric fluid to flush away the molten material. As the machining power increases for higher I_p_, the absorbed power increases, thus, a higher volume of molten material is formed. At the same time, prolonged pulses allow the electro-discharge energy to penetrate deeper into the material, making difficult the efficient flushing^63,64^. In Fig. 10 the successive forming of a thicker and more uniform WL, as the pulse-on time increases, is presented.

Considering the importance to control the AWLT, a correlation between machining conditions and machining results is of extreme interest and practical importance. Again, based on RSM, a semi-empirical model is proposed:

20 $$AWLT = 4.87 + 0.266I_{p} - 0.0115T_{on} - 0.0009I_{p}^{2} + 0.000277T_{on}^{2} + 0.0038I_{p} T_{on}$$

with AWLT in [μm], I_p_ in [A], and T_on_ in [μs].

**Figure 11:**
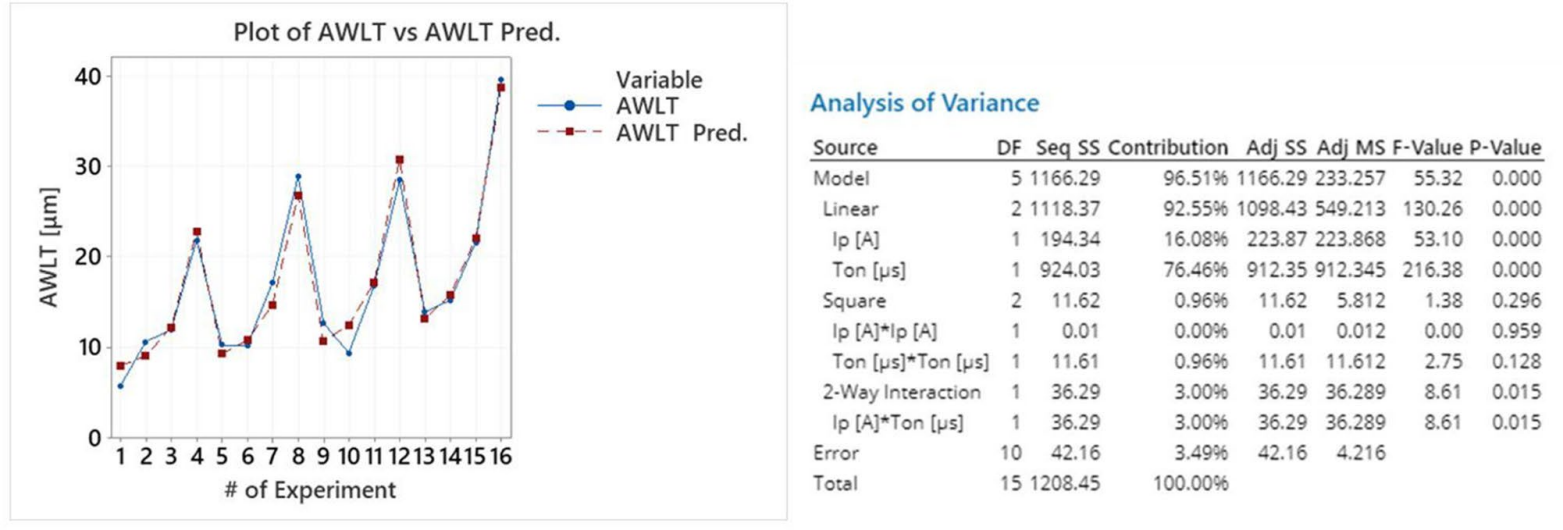
Plot of AWLT vs AWLT predicted and the model’s ANOVA.

The regression fit is regarded as excellent, since the R-sq is over 96.5%, the S value 2.05 and model's P value approximately zero. Moreover, by plotting the AWLT along with model's predictions, see Fig. 11, it is clear that the values have only a limited deviation. Finally, it is worth to point out, that based on the ANOVA table, see Fig. 11, pulse-on current and pulse-on time have both significant contributions, although the contribution of T_on_ is about 450% greater.

### Modeling and simulation-power distribution and plasma flushing efficiency

The numerical simulation provides a useful insight of the process, providing with data and specifics that are extremely difficult or even impossible to be obtained through experiments. Nevertheless, before proceeding in a further analysis, the accuracy and consistency of the model have to verified, in order for the robustness of the deduced conclusions to be ensured. The choice to introduce and base the analysis on "typical spark" was justified previously. Yet, and considering the chaotic nature of EDM^65^, a straight comparison between experimental and simulation results is complicated, involving high risk of error, thus, an indirect verification of the model is more appropriate.

**Figure 12:**
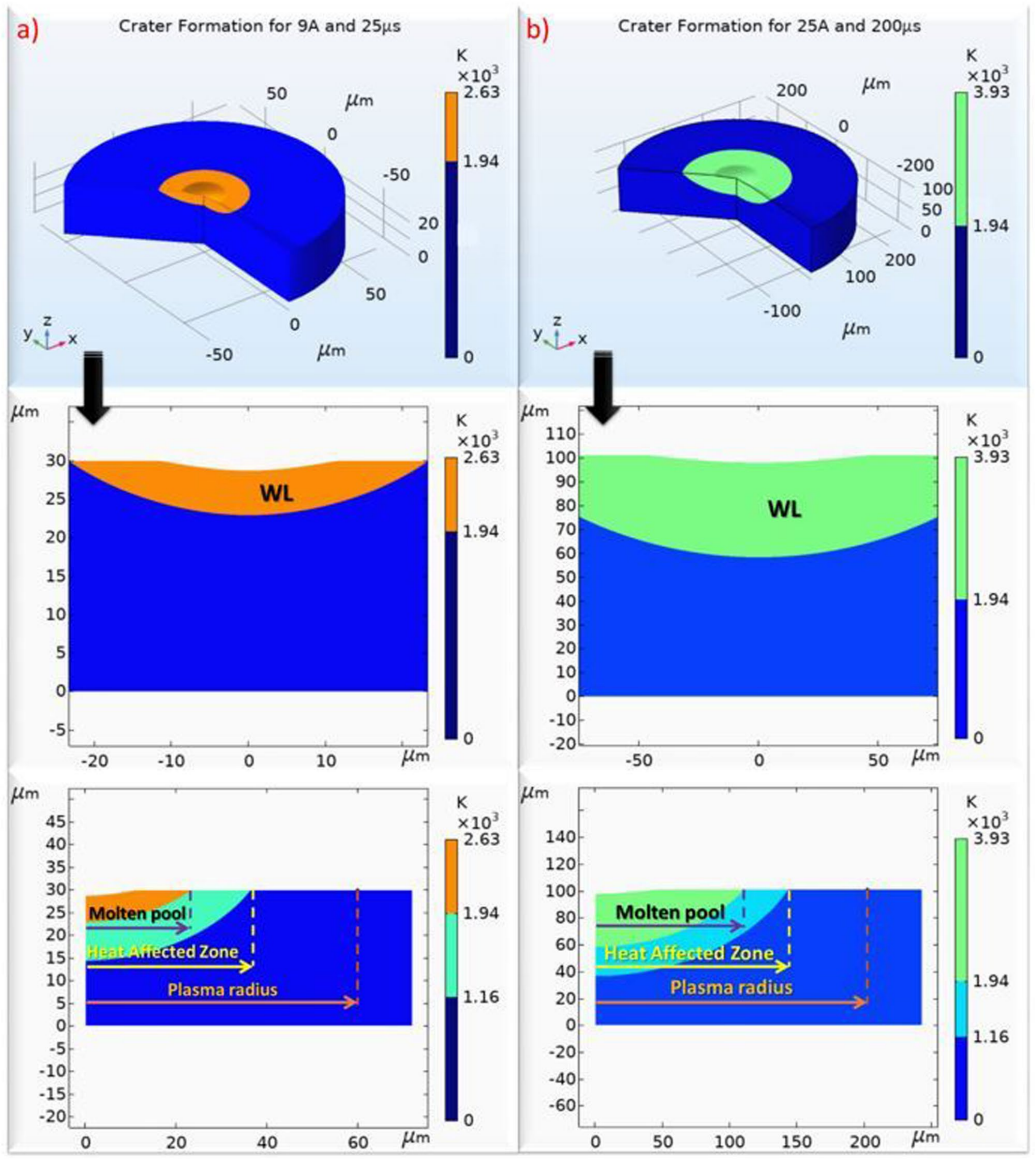
Simulated results for (**a**) 9A and 25 μs, (**b**) 25A and 200 μs where the formatted craters, as well as the WL and Heat Affected areas are depicted.

Since consecutive sparks form a random-like surface, it would be inaccurate to compare the topography of the machined surface with the single crater's simulated geometry. Hence, bibliographic data were used to confirm that the simulated geometry is realistic and that "typical sparks" result to craters that in overlap can potentially form an EDM machined surface. In the studies of Klocke et al.^24^ and Zhang et al.^56^ it can be seen that EDM craters are shallow with high ratio of diameter to depth. In fact, sometimes the WL has almost fully filled the crater's cavity, indicating that very limited material removal occurred^66^. In Fig. 12, the simulated craters for the lowest and highest per pulse discharge energies are depicted. The craters are shallow indeed, while they have a high ratio of diameter to depth. In Fig. 12, the formatted WL is also presented. Since the craters may overlap, the most proper point to estimate the WL thickness is in the crater's center, thus, based on this measurement and assumption, all the subsequent calculations have been made. Obviously, the volume of WL is increased for higher pulse-on current and time. Finally, one last observation that endorses the trusting to the current model is the correlation between the radius of the plasma channel, the heat affected area and the molten area. Kojima et al.^67^ through their experiments concluded that plasma channel diameter is significantly larger than that of the heat affected area, which in turn, is larger than molten area diameter. Practically, the latter means that due to the plasma channel spatial power distribution, there are areas where power density is inefficient to melt the material or even to affect it thermally. The simulation results of the current model, as shown in Fig. 12, are absolutely in line with these findings.

In Table 4. The calculated absorption coefficients for the electrode (F_el_) and the workpiece (F_w_) are listed, as well as the Plasma Flushing Efficiency (PFE), as it is determined in simulations. These coefficients provide an insight of the process, granting a more complete and detailed understanding of the underlying physical mechanisms.

**Table 4:** Absorption coefficients of the electrode (F_el_), and the workpiece (F_w_), and PFE.

#	I_p_ [A]	T_on_ [μs]	F_el_ _(%)_	F_w_ _(%)_	PFE _(%)_
1	9	25	7.10	5.1	3.8
2	9	50	7.60	7.1	2.1
3	9	100	9.30	7.8	2.5
4	9	200	10.00	11.1	1.1
5	13	25	8.10	6.7	2.1
6	13	50	9.40	6.5	3.8
7	13	100	10.00	8.8	2.1
8	13	200	11.00	12.4	1.1
9	17	25	7.50	7.5	1.2
10	17	50	8.70	5.8	4.5
11	17	100	9.80	8.1	2.4
12	17	200	11.00	11.3	1.5
13	25	25	7.10	7.5	0.9
14	25	50	8.40	7.2	1.9
15	25	100	9.60	8.8	1.8
16	25	200	10.80	13.9	1.0

**Figure 13:**
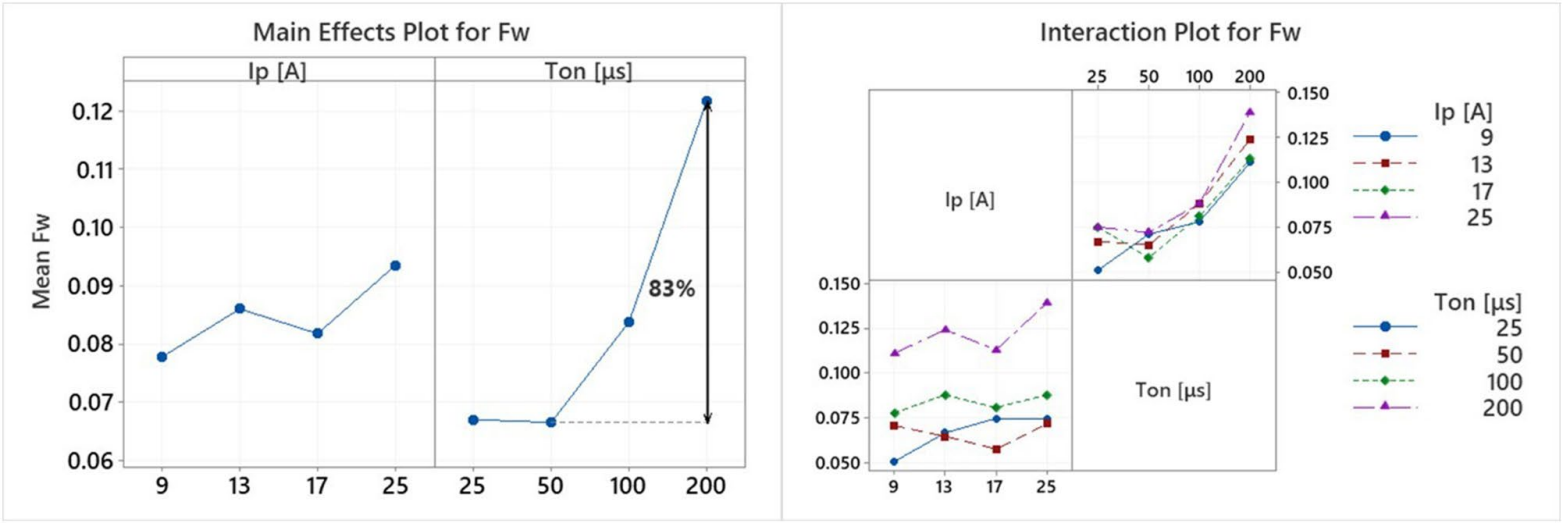
Main effects plot and interaction plot of F_w_.

The energy absorption by the workpiece is definitely related to the machining efficiency and the MRR. Nevertheless, its correlation is quite different from the expected, explainable but thorny. In Fig. 13, the Main Effects Plot and the Interaction Plot for F_w_ are presented.

Seemingly, the pulse-on time has a clear effect on the F_w_, while I_p_ has a more vague one. More specifically, an increase in pulse-on time results to an increase in energy absorption by the workpiece of approximately 83%, while an increase in pulse-on current has an insignificant effect, as F_w_ slightly increases or decreases. At first glance, this seems paradoxical and opposite with the above conclusions regarding MRR, since in MRR the I_p_ has the major effect. However, the interpretation lies on the understanding of the undergoing heat transfer mechanism. Indeed, as the T_on_ increases, a more expanded plasma channel is formed, hence, the heat transfer is facilitated, thus, increased amount of energy is absorbed by the workpiece. On the other hand, higher pulse-on current, i.e., higher machining power and power density, seems not to have any particular favorable or negative effect on energy absorption. The reasons why the higher energy absorption does not lead to higher MRR are the WL and the flushing capabilities. As there are limited flushing capabilities, greater volumes of molten material form thicker WL, thus, the increased absorbed energy is consumed by the melting and re-melting of the thick formed WL, and does not increase MRR. The aforementioned explanation is evidenced by the following analysis of PFE, where the PFE is significantly decreased for more intense machining parameters. The estimation of F_w_ is important and useful, not only to accurate develop models and simulations, but as an index of machining efficiency in respect to the machining parameters. Thus, based in RSM, a correlation of F_w_ with pulse-on current and time is proposed:

21 $$F_{w} = 0.03991 + 0.000864I_{p} + 0.00331T_{on}$$

with I_P_ in [A] and T_on_ in [μs].

In the current model, only the linear terms were necessary to be included, with the T_on_ having over 58% contribution on the Seq SS. The fit considered sufficient, as R-sq is over 90%, S value 0.0081, and model's P value almost zero. This semi-empirical relation is considered that can be employed in modeling single EDM sparks for titanium and titanium alloys with similar thermophysical properties.

**Figure 14:**
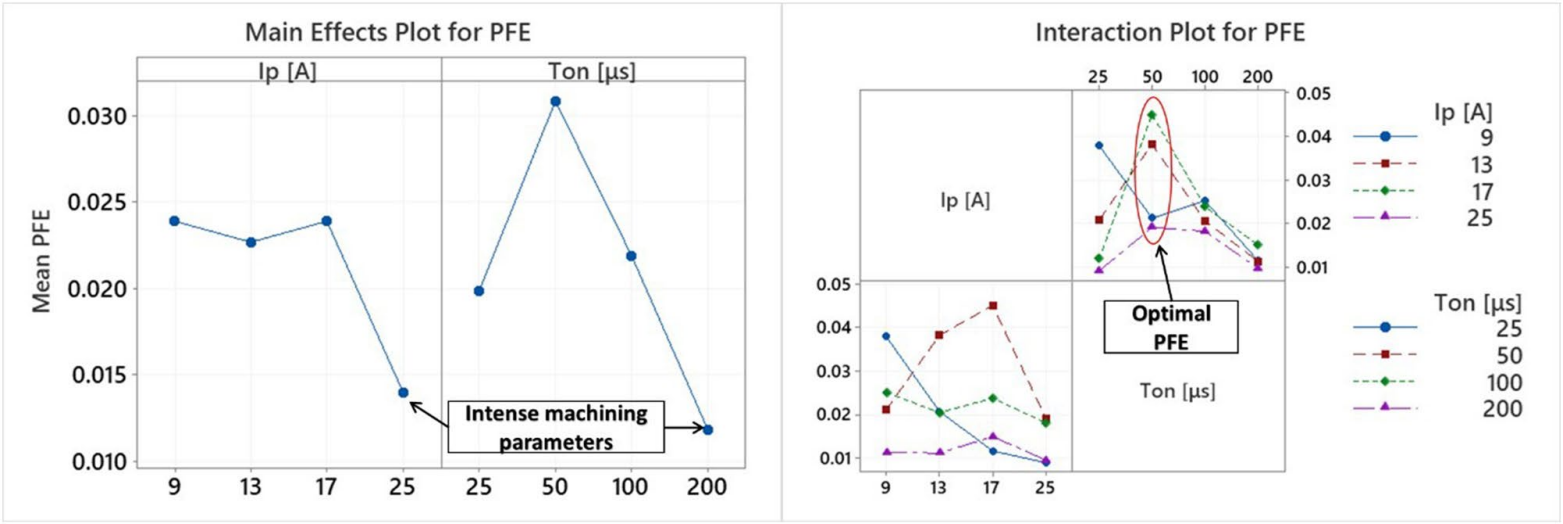
Main effects plot and interaction plot of PFE.

As was previously mentioned, PFE is a decisive parameter for process efficiency, and attainment of high MRR. The PFE depends on machining conditions, namely pulse current, pulse duration, flushing method, and is related to the AWLT. Insufficient flushing, i.e., lower PFE, creates thicker WL, affecting the process in overall. In Fig. 14, the Mean Effects Plot and the Interaction Plot of PFE are depicted. On the contrary with the former examined machining indexes, PFE correlation to the machining parameters is not clear. Nevertheless, some useful conclusions can be deduced; specifically, for the most intense machining parameters the lowest mean PFE was estimated, while for most pulse-on currents, the higher PFE was observed for pulses at 50 μs. These observations denoted that intense machining conditions lack of efficiency in power consumption, while, at the same time, there is an optimal set of parameters to maximize PFE.

Since multiple factors affect the overall process feasibility and efficiency, e.g., TWR, and/or SQ requirements, the optimal machining conditions have to be carefully picked, as the case may be. Taking in mind all the above mentioned, regarding the utility to estimate accurately the PFE, based on RSM, a semi empirical correlation is proposed:

22 $$\begin{aligned} & PFE = 0.0360 + 0.00314I_{p} + 256 \cdot 10^{ - 6} T_{on} - 528 \cdot 10^{ - 6} AWLH - 0.000021I_{p}^{2} - 2 \cdot 10^{ - 6} T_{on}^{2} \\ & \quad + 81 \cdot 10^{ - 6} AWLT^{2} + 17 \cdot 10^{ - 6} I_{p} T_{on} - 149 \cdot 10^{ - 6} I_{p} AWLT + 9 \cdot 10^{ - 6} T_{on} AWLT \\ \end{aligned}$$

with I_P_ in [A] , T_on_ in [μs] and AWLT in [μs].

**Figure 15:**
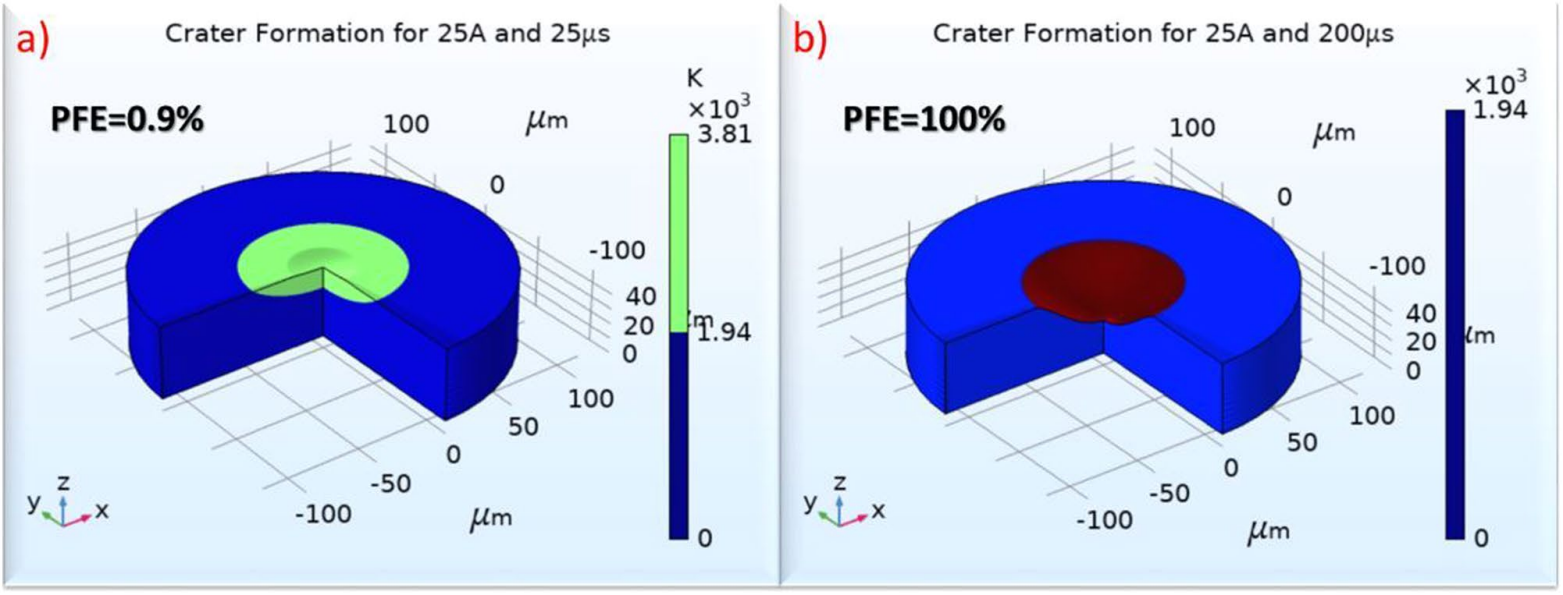
Craters formation for 25A and 25 μs with (**a**) PFE equals to experimental measured (0.9%), and (**b**) PFE = 100% (theoretical maximum with no WL formation).

The model is adequately fitted to the data, having R-sq over 94%, S value less than 0.004, and P value 0.004. An important highlight at this point is that for PFE, for the regression model, apart from the pulse-on current and time that until now were employed, the AWLT was also integrated into the equation. The terms that include the AWLT contribute in Seq SS over 50%, implying and statistically prove the close relation between the PFE and the WL formation. In Fig. 15, it is juxtaposed the material removal with a low PFE, Fig. 15a with PFE = 0.9%, and with a theoretical PFE 100%, Fig. 15b, without the formation of WL. A PFE of 100% is certainly unrealistic, but this comparison gives a notion regarding the potential of improvement in MRR, utilizing a more effective flushing of the molten material, and thus, increasing the PFE.

**Figure 16:**
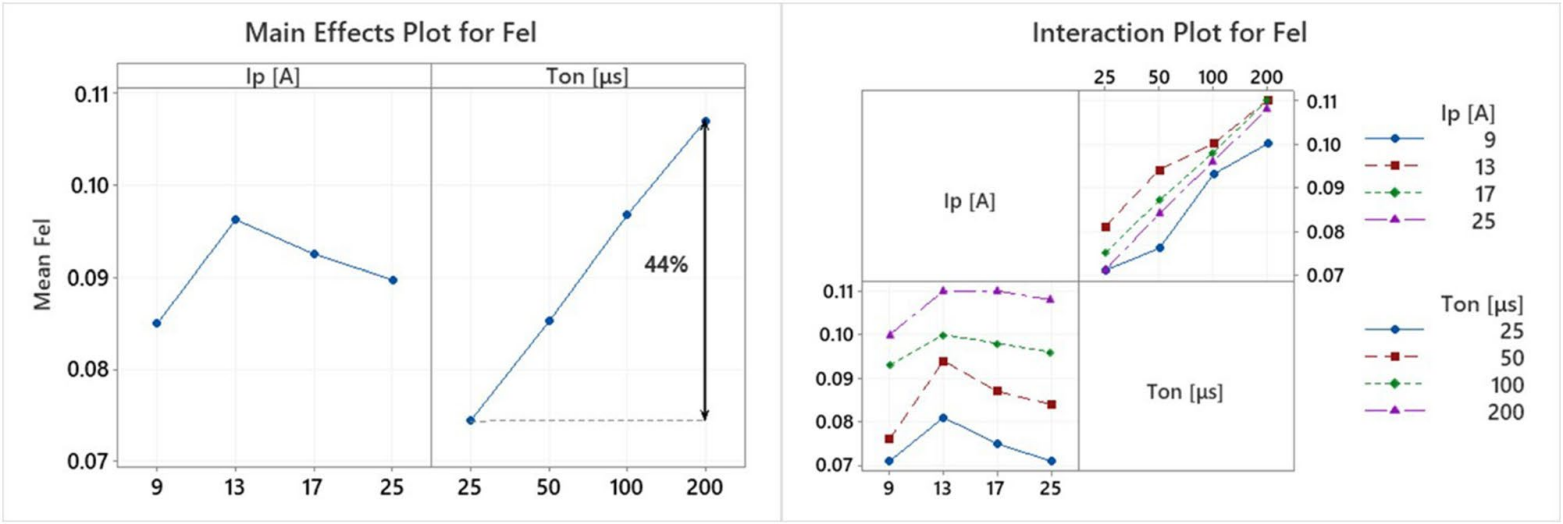
Main effects plot and interaction plot of F_el_.

The final process parameter that will be analyzed is the power absorption coefficient of the electrode (F_el_). It is an interesting and important index, which can provide a clue about electrode’s erosion mechanism, and hence, the TWR. In Fig. 16, the Main Effects Plot and the Interaction Plot of F_el_ are presented.

**Figure 17:**
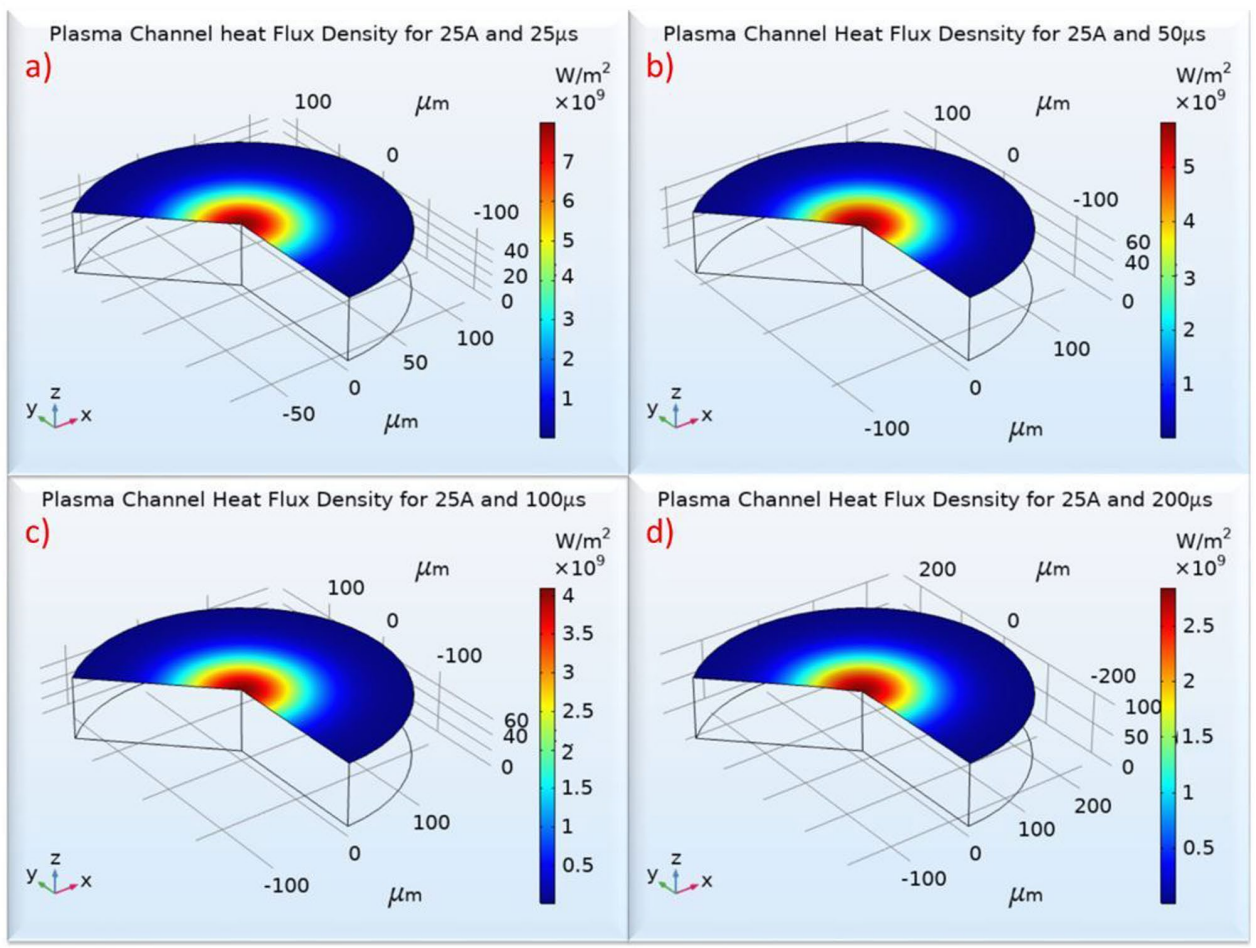
Electrode’s Plasma Channel’s Heat Flux for 25A and (**a**) 25 μs, (**b**) 50 μs, (**c**) 100 μs and (**d**) 200 μs.

F_el_ follows similar pattern with F_w_, namely, changes in pulse-on current seems to have a fuzzy and trifling impact, while the change in pulse-on time has a clear effect. The increase of F_el_ in respect to pulse duration is almost linear, for all the pulse-on currents; the mean F_el_ increased approximately by 44% between 25 and 200 μs T_on_. The improvement of TWR for higher T_on_ can be partially attributed to this change. Although more energy is absorbed by the electrode, causing additional wear, the F_w_ is increased in almost double rate compared with F_el_, see Fig. 13, thus, it may subsist increased wear, but, at the same time, increased material removal occurs, limiting in such a way the TWR. The second causation of lower TWR for high T_on_s is the power distribution, a conclusion that can be justified by the simulation results. Although with an increase in pulse-on time, higher proportion of energy is absorbed by the electrode, the power is distributed in a wider area, since the plasma channel radius increases, thus, the power density is significantly affected. This reduction is depicted in Fig. 17, where the plasma channel's heat flux density is gradually decreased as T_on_ increases.

**Figure 18:**
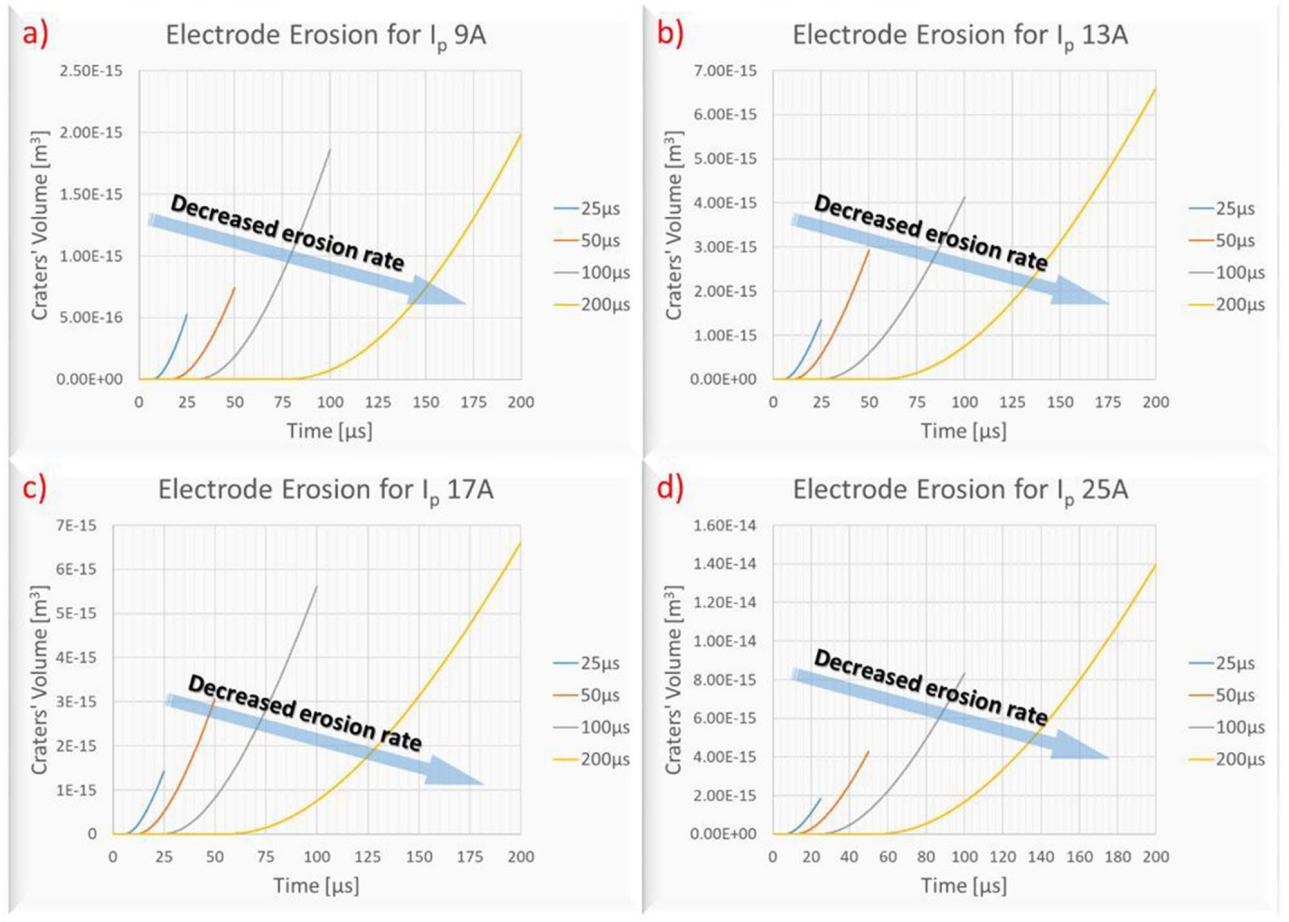
Erosion rates for different machining parameters for (**a**) 9A, (**b**) 13A, (**c**) 17A and (**d**) 25A.

The above-mentioned mechanisms result to a decrease in electrode's erosion rate and a delay in erosion initiation as T_on_ increases. This can characteristically be observed in the plots of Fig. 18, where for the same pulse-on current, the simulated erosion for higher pulse-on times is more "smooth" and delayed favoring a lower TWR.

Finally, by employing the RSM, a correlation between the F_el_ and the machining parameters is proposed:

23 $$F_{el} = 0.0365 + 0.00388I_{p} + 0.000399T_{on} - 0.000115I_{p}^{2} - 1 \cdot 10^{ - 6} T_{on}^{2} + 2 \cdot 10^{ - 6} I_{p} T_{on}$$

with I_P_ in [A] , and T_on_ in [μs]. The regression model is adequately fitted on the data, having R-sq over 94%, S value less than 0.004, and P value almost zero.

**Figure 19:**
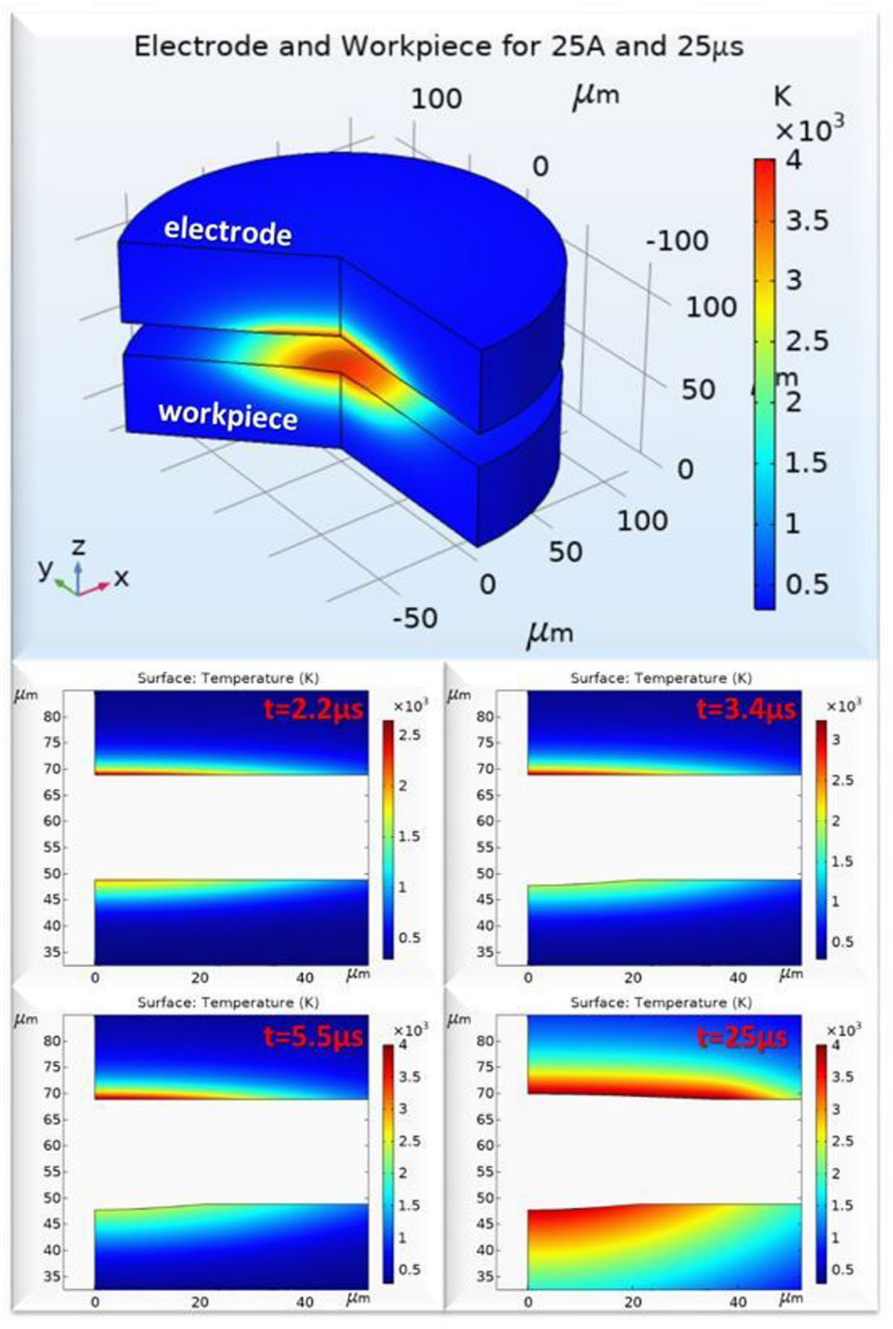
Electrode and workpiece erosion simulation.

In closing, the concurrent erosion of electrode and workpiece is presented in Fig. 19, through some characteristic snapshots of the simulation. More specifically:

- At 2.2 μs the erosion of the workpiece starts, while the electrode's high sublimation temperature delays the electrode's erosion initiation.
- At 3.4 μs the crater on the workpiece has been fully formed, and the material is overheated, forming the WL.
- The temperature on the electrode reaches the sublimation limit at 5.5 μs, when the electrode erosion starts, with the delay that previously was mentioned.
- At 25 μs, in the end of the pulse, the two craters have been formed, observing that due to the differences in materials' thermo-physical properties and the material removal mechanism (ablation vs melting), the electrode's crater is wider and shallower, while higher temperature gradients are developed in electrode due to its lower thermal conductivity.

## Conclusions

In the current study, the machining of Titanium Grade2 with EDM was investigated, by combining a series of experiments with modeling, aiming to an insight view of the process. A full-scale experimental procedure was conducted, with control parameters the pulse-on current and pulse-on time, using graphite electrode. The MRR, TWR and AWLT were calculated as the major machining performance indexes. Moreover, a heat transfer numerical model with coupled deformed geometry was developed, in order to accurately and realistically simulate the spark erosion mechanism and to estimate parameters that cannot be obtained through experiments. Namely, the power distribution between electrode and workpiece was estimated, as well as the plasma flushing efficiency. For all the above-mentioned process indexes ANOVA was performed, while the Response Surface Methodology was employed in order to define the correlation between machining parameters and results, and to propose respective semi-empirical relations. Finally, emphasis was placed on the interpretation of the results, based on the undergoing physical mechanisms. In brief, the deduced conclusions of the current study are:

- The MRR is mainly affected by the pulse-on current, with an increase of I_p_ resulting to a higher MRR. The pulse-on time affects MRR in an ambiguous way, since there is an upper limit of MRR for a certain T_on_, and further increase has insignificant or negative result on MRR.
- The TWR has a major dependence on pulse-on time, with an increase in pulse duration resulting to lower TWR. On the other hand, variations in I_p_ have a fuzzy influence on TWR. The TWR is a percentage comparative index that indicates the ratio of material removal between the electrode and the workpiece, thus, it should not be straightly compared with MRR. TWR is an index of process’ efficiency in term of electrode consumption rate, and based on the experimental results, it is deduced that it increases for higher pulse-on times.
- The AWLT is increased for more intense machining parameters, i.e., for higher pulse-on currents and times. The mean value of AWLT increased by 80% and 197.5% as the I_p_ and T_on_ increased from 9A to 25A and from 25 to 200 μs, respectively. The melted material volume increases as more intense machining parameters are employed, and hence, the re-solidified material volume is increased, too, forming a thicker WL.
- The absorption coefficients F_el_ and F_w_ increase for higher pulse-on times, while pulse-on current seems to only slightly affect them.
- As EDM is a multi-parameter and complex process, factors like the power density distribution, materials’ thermo-physical properties, flushing efficiency and combination of machining parameters have always to be considered in production planning, to optimize and render it as a feasible alternative.
- Employing the proposed correlations about MRR, TWR and AWLT, an optimal machining planning can be achieved, as the case may be, saving time and resources. At the same time, modeling consists a robust analysis method, with realistic and accurate results, rendering simulation as a powerful tool in research for improving and optimizing the EDM process. Starting from the machining conditions and by following the presented methodology, a comprehensive and detailed view of the process and its results can be obtained.

## Data Availability

The datasets generated and analyzed during the current study are available from the corresponding author on reasonable request.
